# Citraconate inhibits ACOD1 (IRG1) catalysis, reduces interferon responses and oxidative stress, and modulates inflammation and cell metabolism

**DOI:** 10.1038/s42255-022-00577-x

**Published:** 2022-06-02

**Authors:** F. Chen, W. A. M. Elgaher, M. Winterhoff, K. Büssow, F. H. Waqas, E. Graner, Y. Pires-Afonso, L. Casares Perez, L. de la Vega, N. Sahini, L. Czichon, W. Zobl, T. Zillinger, M. Shehata, S. Pleschka, H. Bähre, C. Falk, A. Michelucci, S. Schuchardt, W. Blankenfeldt, A. K. H. Hirsch, F. Pessler

**Affiliations:** 1 Research Group Biomarkers for Infectious Diseases, Helmholtz Centre for Infection Research, Braunschweig, Germany; 2 Research Group Biomarkers for Infectious Diseases, TWINCORE Centre for Experimental and Clinical Infection Research, Hannover, Germany; 3 Helmholtz Institute for Pharmaceutical Research Saarland – Helmholtz Centre for Infection Research, Saarbrücken, Germany; 4 Department of Structure and Function of Proteins, Helmholtz Centre for Infection Research, Braunschweig, Germany; 5 Neuro-Immunology Group, Department of Cancer Research, LIH Luxembourg Institute of Health, Luxembourg, Luxembourg; 6 Faculty of Science, Technology and Medicine, University of Luxembourg, Esch-Belval, Luxembourg; 7 Division of Molecular Medicine, University of Dundee, Dundee, UK; 8 Fraunhofer Institute for Toxicology and Experimental Medicine (ITEM), Hannover, Germany; 9 Institute of Clinical Chemistry and Clinical Pharmacology, University Medical Centre Bonn, Bonn, Germany; 10 Institute of Immunology, Philipps-University Marburg, Marburg, Germany; 11 Institute of Medical Virology, Justus-Liebig-University Giessen, Giessen, Germany; 12 National Research Centre, Giza, Egypt; 13 German Center for Infection Research partner site Giessen, Giessen, Germany; 14 Research Core Unit Metabolomics, Hannover Medical School, Hannover, Germany; 15 Department of Transplantation Immunology, Hannover Medical School, Hannover, Germany; 16 Luxembourg Centre for Systems Biomedicine, University of Luxembourg, Esch-Belval, Luxembourg; 17 Institute for Biochemistry, Biotechnology and Bioinformatics, Technische Universität Braunschweig, Braunschweig, Germany; 18 Department of Pharmacy, Saarland University, Saarbrücken, Germany; 19 Centre for Individualised Infection Medicine, Hannover, Germany

**Keywords:** Metabolomics, Applied immunology, Metabolism, Immunology

## Abstract

Although the immunomodulatory and cytoprotective properties of itaconate have been studied extensively, it is not known whether its naturally occurring isomers mesaconate and citraconate have similar properties. Here, we show that itaconate is partially converted to mesaconate intracellularly and that mesaconate accumulation in macrophage activation depends on prior itaconate synthesis. When added to human cells in supraphysiological concentrations, all three isomers reduce lactate levels, whereas itaconate is the strongest succinate dehydrogenase (SDH) inhibitor. In cells infected with influenza A virus (IAV), all three isomers profoundly alter amino acid metabolism, modulate cytokine/chemokine release and reduce interferon signalling, oxidative stress and the release of viral particles. Of the three isomers, citraconate is the strongest electrophile and nuclear factor-erythroid 2-related factor 2 (NRF2) agonist. Only citraconate inhibits catalysis of itaconate by *cis*-aconitate decarboxylase (ACOD1), probably by competitive binding to the substrate-binding site. These results reveal mesaconate and citraconate as immunomodulatory, anti-oxidative and antiviral compounds, and citraconate as the first naturally occurring ACOD1 inhibitor.

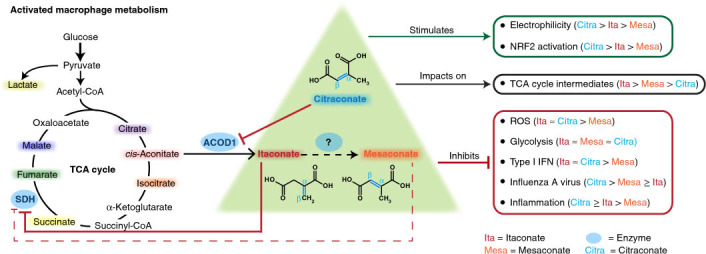

## Abstract (web summary)

The immunomodulatory effects of mesaconate and citraconate, naturally occurring isomers of the immunomodulatory metabolite itaconate, are investigated. Mesaconate is shown to be derived from itaconate, and citraconate is shown to inhibit ACOD1 and display stronger NRF2 agonism.

## Main

**Fig. 1:**
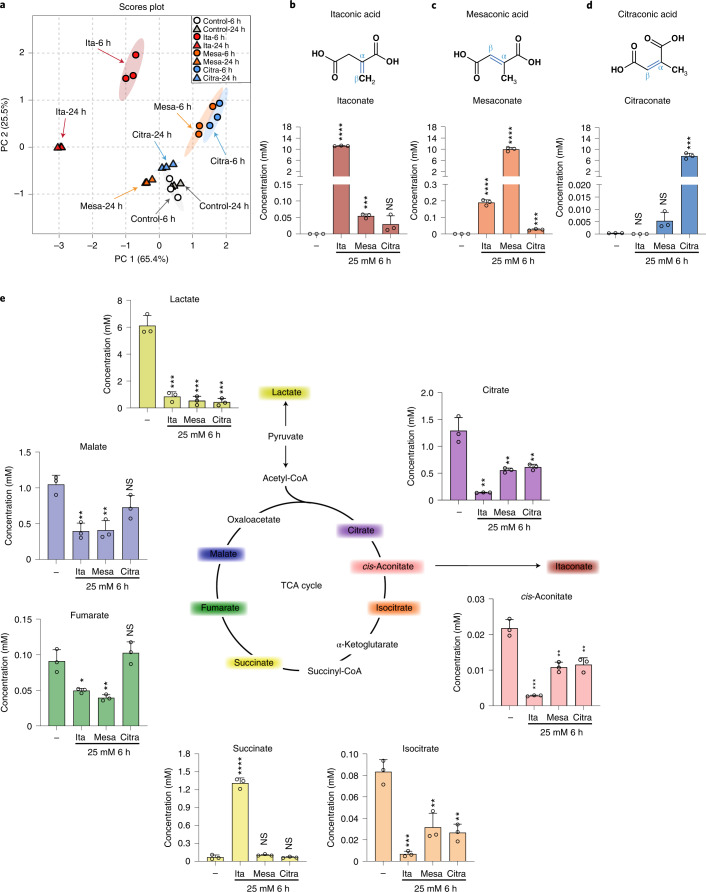
Differential impact of itaconate isomers on concentrations of selected TCA cycle intermediates and lactate in dTHP1 cells. Selected TCA intermediates and lactate were measured by HPLC–MS/MS at 6 and 24 h in the itaconate isomer uptake experiments shown in Extended Data Fig. 1. Concentrations are expressed based on calculated cell volume. **a**, PCA illustrating strong alterations due to all three isomers at 6 h, but a relative normalization of mesaconate and citraconate effects by 24 h. **b**–**d**, The measured isomer is indicated above each graph, the added isomers below the *x* axis. **e**, Concentrations of lactate and selected TCA intermediates (concentrations given on the *y* axes) 6 h after addition of the isomers indicated below the *x* axis. All three isomers reduce lactate levels (consistent with inhibition of glycolysis), but only itaconate raises succinate levels, suggesting inhibition of SDH. Citra, citraconic acid; Ita, itaconic acid; Mesa, mesaconic acid. *n* = 3 biological replicates, means ± s.d. Unpaired *t*-test. *P ≤ 0.05, **P ≤ 0.01, ***P ≤ 0.001, ****P ≤ 0.0001; NS, not significant.

The small unsaturated dicarboxylic acids itaconic, mesaconic and citraconic acid are naturally occurring isomers that differ only by the location of a double bond (Fig. 1). Itaconate is a key metabolite of activated macrophages and is the product of the mitochondrial enzyme ACOD1^1,2^. It is being intensely investigated as a link between metabolism and immunity, has immunomodulatory properties (reviewed in refs. ^3,4^) and has been detected in human biofluids, cells and tissues in a variety of inflammatory and infectious diseases as well as in rodent models of human diseases (summarized in ref. ^5^). Information about the origin and catabolism of mesaconate and citraconate in eukaryotes is sparse. Considering their high structural similarity, interconversion between the three isomers is conceivable, for instance via endogenous isomerases. Based on work on itaconate metabolism using isolated liver mitochondria^6^, Nemeth et al. suggested that mesaconate may be a product of itaconate catabolism via itaconyl-CoA^7^. The only study suggesting a biosynthetic mechanism of citraconate in higher organisms is based on metabolite profiling of patients with methylmalonic acidaemia, where it was postulated to be a catabolite of the branched chain amino acid (BCAA) isoleucine^8^. There is growing evidence that increased levels of mesaconate or citraconate can be associated with human metabolic diseases (summarized in ref. ^5^). However, it remains to be clarified whether these increased concentrations are pathophysiologically relevant or whether they merely represent epiphenomena of derangements in metabolism. In humans, there is no information about the tissue distribution of mesaconate or citraconate. However, in a screen of organs from healthy mice, we have recently shown that mesaconate occurs in lymph nodes, and citraconate in lymph nodes and spleen, raising the possibility of some function in immunity^5^.

The immunomodulatory and cytoprotective properties of itaconate and chemically modified derivatives such as dimethyl- and 4-octyl-itaconate (4-OI) are being studied intensely to create therapeutic interventions for inflammatory and degenerative diseases^3,4^ and viral infections^9,10^. By contrast, it is not known whether exogenously applied mesaconate or citraconate can be taken up by cells and whether they exert immunomodulatory, anti-oxidative or anti-infective effects similar to itaconate. Beyond the well-documented roles of itaconate in immunometabolism, aberrant synthesis of itaconate by ACOD1 has been implicated in carcinogenesis. Itaconate that is released from peritoneal macrophages may serve as a growth factor for tumours that have spread into the peritoneal cavity^11^, and a protumorigenic role of ACOD1 has also been reported in gliomas^12,13^. ACOD1 inhibitors may therefore have anti-neoplastic properties, but it is not known whether there are endogenous molecules that inhibit ACOD1 activity. Considering these open questions, we employed a combination of cell-free assays, cellular models, an IAV infection model and a mouse model of lipopolysaccharide (LPS)-induced inflammation to: (1) test whether there is interconversion among the three isomers; (2) assess the impact of mesaconate and citraconate on cell metabolism, inflammation, oxidative stress and infectivity of IAV; and (3) test whether any of the three isomers inhibit ACOD1 activity.

## Results

### Absence of direct conversion of itaconate to its isomers

Searching for the origins of mesaconate and citraconate, we first tested whether there is spontaneous interconversion among the three isomers and whether ACOD1 can also catalyse mesaconate or citraconate synthesis. There was no evidence of mesaconate or citraconate synthesis in a cell-free ACOD1 enzyme assay^2^ (Supplementary Fig. 1b) and there was no spontaneous interconversion when pure compounds were incubated in RPMI medium for up to 24 h (Supplementary Fig. 1c–e). However, biologically irrelevant low contaminating concentrations of each isomer were detected in pure stocks of the respective other isomers (Supplementary Fig. 1g).

### Efficient uptake of the isomers into dTHP1 cells

**Extended Data Fig. 1:**
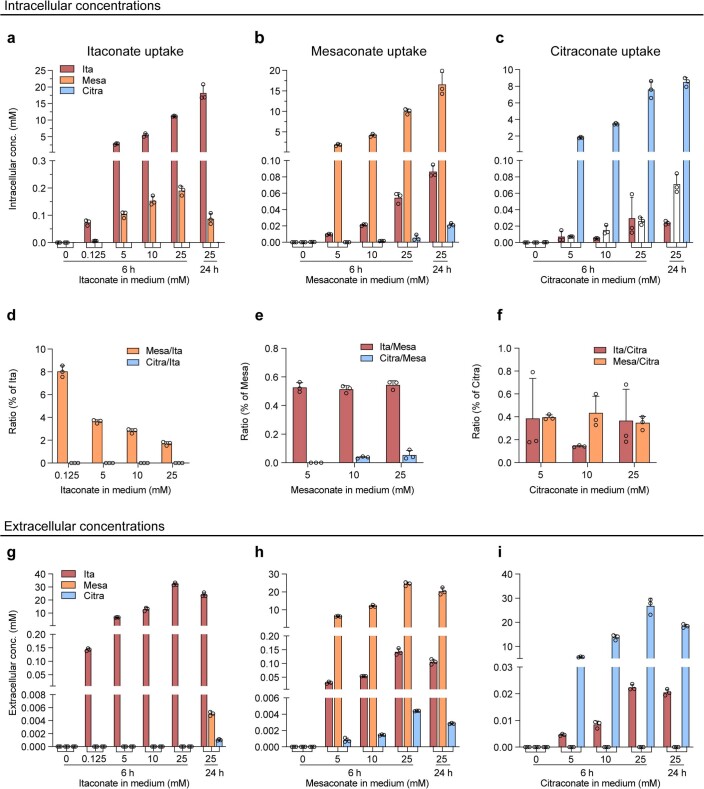
Efficient uptake of all three isomers into dTHP1 cells. Cells were incubated with 25 mM of the respective isomer for 6 and 24 h and concentrations of the three isomers were then measured by HPLC-MS/MS in supernatants and extracts of washed cells. a–f, Intracellular concentrations. Absolute concentrations are shown in a–c, and fractions of the other two isomers with respect to the isomer added to the medium in d–f. There is a dose-dependent increase in mesaconate when itaconate is added to medium (g) but a decrease in the mesaconate/itaconate fraction with increasing itaconate concentrations (d). Both observations are consistent with intracellular conversion of a small fraction of itaconate to mesaconate by a saturable, presumably enzymatic, process. g–i, Itaconate concentrations remain stable in cell supernatants. The known impurities of itaconate in mesaconate and citraconate are detected again (see also Supplementary Fig. S1c–g). *n*=3 biological replicates, means ±SD.

Cordes et al. showed that exogenously added itaconate (25 mM) can lead to intracellular concentrations of 14 mM in resting murine RAW 264.7 cells^14^. After determining non-toxic concentrations of the isomers (Supplementary Fig. 1h), we tested their uptake into differentiated human macrophage-like cells (dTHP1). Indeed, all three isomers were taken up efficiently (Extended Data Fig. 1a–f) and there was no evidence of de novo synthesis of citraconate or itaconate. However, when itaconate was added to the medium, mesaconate appeared intracellularly. The concentration of mesaconate with respect to intracellular itaconate ranged from 1.7% to 8.0% and correlated inversely with intracellular itaconate concentration (Extended Data Fig. 1d).

### Mesaconate accumulation depends on itaconate synthesis

**Extended Data Fig. 2:**
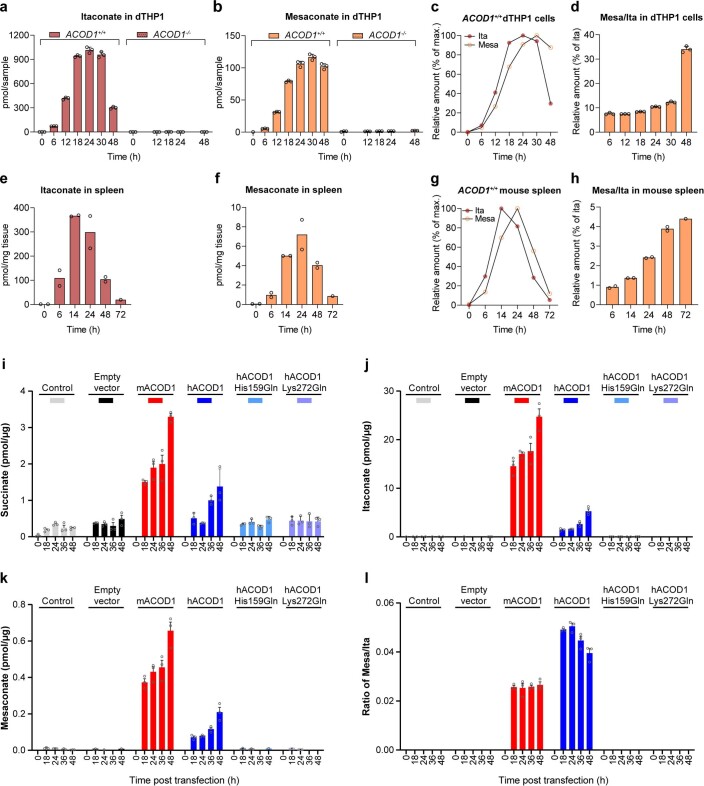
Dependence of mesaconate accumulation on itaconate synthesis. a–h, Mesaconate accumulation peaks after itaconate accumulation in LPS-induced inflammation and depends on prior itaconate synthesis in dTHP1 cells. a–d, dTHP1 cells were stimulated with LPS/IFNγ and intracellular concentrations of the three isomers were measured by HPLC-MS/MS at the indicated time points. Citraconate was not detected. Neither itaconate nor mesaconate were detected in *ACOD1*^–/–^ dTHP1 cells. The maximal itaconate concentration at 24 h was 460 µmol/L. n=3 biological replicates, means ±SD. e–h, Systemic inflammation was elicited in C57BL/6N mice by intraperitoneal LPS injection, and concentrations of the three isomers in spleen homogenates were measured by HPLC-MS/MS at the indicated time points. n=2 mice per time point. i–l, Mesaconate accumulation in A549 cells overexpressing ACOD1. Human or murine ACOD1 was expressed in A549 cells by transient transfection, and concentrations of the three isomers as well as selected TCA intermediates and lactate were measured by HPLC-MS/MS at the indicated time points. Values <LLOQ were considered missing values. Transfection of both enzymes resulted in a parallel rise of itaconate and mesaconate concentrations, whereas citraconate was not detected. mACOD1 expression led to a greater increase in succinate levels, consistent with greater SDH inhibition due to higher itaconate synthesis when compared to hACOD1. His159Gln and Lys272Gln are inactive hACOD1 variants^1^. Concentrations are expressed per µg protein. n=3 biological replicates, mean ±SD.

Of note, a small amount of mesaconate also appeared in supernatants 24 h after addition of itaconate, consistent with the secretion of intracellularly generated mesaconate (Extended Data Fig. 1g). We therefore tested whether endogenously synthesized itaconate would also lead to mesaconate accumulation. Indeed, LPS/interferon-γ (IFN-γ) stimulation of dTHP1 cells led to mesaconate accumulation that peaked after itaconate (Extended Data Fig. 2a–d), whereas neither itaconate nor mesaconate was detectable in *ACOD1*^*−/−*^ dTHP1 cells stimulated with LPS/IFN-γ. Citraconate was not detected in either cell type. An apparent conversion of itaconate to mesaconate was also detected in spleen from mice during a 72 h time course of LPS-induced systemic inflammation (Extended Data Fig. 2e–h). However, the fractional conversion of itaconate to mesaconate was only about 4%, which is much less than in dTHP1 cells. To test whether the conversion of itaconate to mesaconate can also take place in cells not physiologically expressing ACOD1, we transfected the respiratory epithelial cell line A549 with a vector constitutively expressing human ACOD1 (hACOD1) or murine ACOD1 (mACOD1; Extended Data Fig. 2i–l). In contrast to the delay observed in dTHP1 cells, there was a parallel increase in itaconate and mesaconate, with mesaconate amounting to 4%–5% (hACOD1) and 2.5%–2.7% (mACOD1) with respect to itaconate, but absolute mesaconate concentrations were much higher in cells expressing mACOD1, which produce far higher levels of itaconate than the human enzyme^2^ (Extended Data Fig. 2j,k). Citraconate was not detected. Itaconate can inhibit SDH, thereby raising succinate levels^15^. Indeed, succinate accumulated markedly in cells expressing mACOD1 (Extended Data Fig. 2i). A smaller increase in succinate was observed in hACOD1-transfected cells, but only at itaconate concentrations that were higher than baseline succinate levels. Taken together, the results shown in Extended Data Figs. 1 and 2 suggest that citraconate has an itaconate-independent origin, whereas mesaconate originates from itaconate via an enzyme-mediated process that occurs physiologically at least in spleen and macrophages, but is not strictly cell-type dependent.

### Effects of the isomers on lactate and tricarboxylic acid cycle intermediates

**Extended Data Fig. 3:**
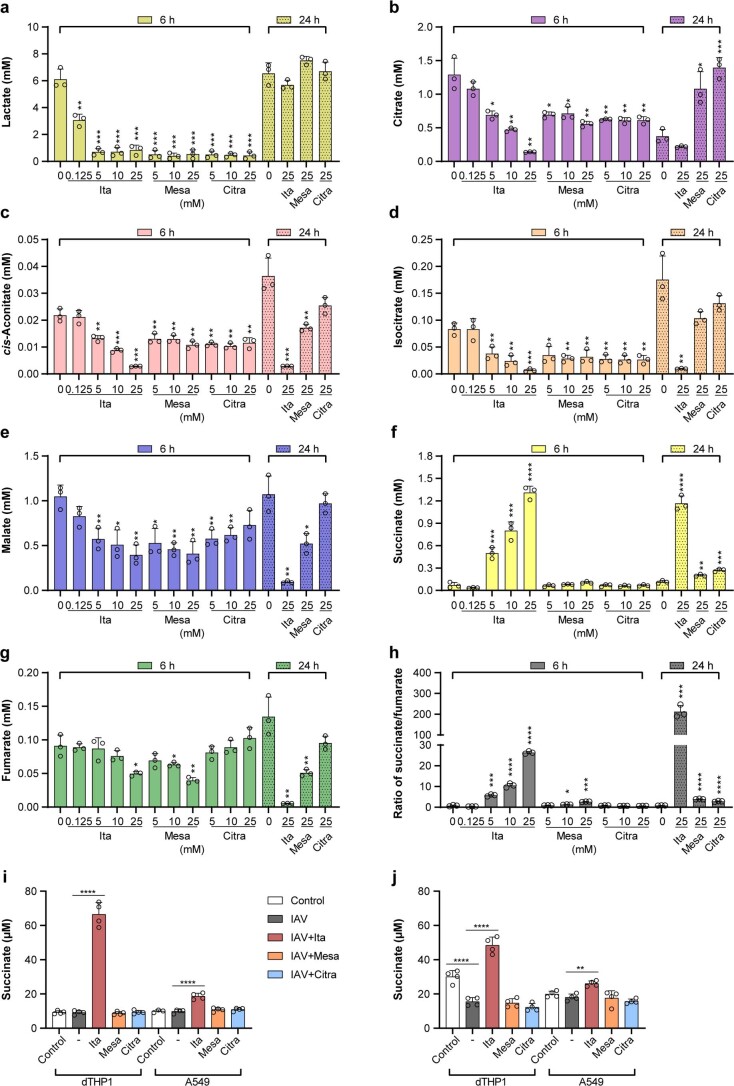
Common and unique effects of itaconate, mesaconate, and citraconate on intracellular levels of lactate and TCA intermediates. Analysis based on the experiment shown in Fig. 1, showing the 24 h time point in addition. The 0.125 mM concentration of itaconate was used to mimic the maximal contamination of itaconate found in 25 mM mesaconate. a–g, Concentrations of the indicated analytes 6 h after treatment with 5, 10, or 25 mM, and 24 h after treatment with 25 mM. h, Ratio of succinate to fumarate, indicating SDH inhibition. *n*=3, mean ±SD. Unpaired T-test. i,j, Succinate levels in IAV-infected dTHP1 and A549 cells, obtained in the same experiment as shown in Extended Data Fig. 5d,e and an independent experiment. Only itaconate addition raises succinate levels, and the effect is more pronounced in dTHP1 than in A549 cells. *n*=4 biological replicates, mean ±SD. One-way ANOVA followed by Dunnett’s multiple comparisons test. P values: * ≤0.05, ** ≤0.01, *** ≤0.001, **** ≤0.0001.

In addition to inhibiting SDH, itaconate can shift central glucose metabolism from aerobic glycolysis to the pentose-phosphate shunt^16^. We therefore assessed the effects of adding increasing concentrations of the three isomers on lactate, succinate and other selected tricarboxylic acid (TCA) cycle intermediates. As shown in the principle component analysis (PCA) plot in Fig. 1a, the effects of the isomers differed markedly 6 h after addition and were greatest for itaconate. Of note, by 24 h, changes due to itaconate increased further, whereas those due to mesaconate and citraconate began to normalize (Fig. 1a). All three isomers markedly reduced lactate concentrations at 6 h (Fig. 1e), but the effect abated by 24 h (Extended Data Fig. 3a). Remarkably, there was a unique dose-dependent increase in succinate at both time points after addition of itaconate, consistent with SDH inhibition, whereas after adding mesaconate or citraconate, succinate levels increased slightly only by 24 h. However, when using the succinate/fumarate ratio as a measure of SDH inhibition, weak inhibition was also evident at high doses of mesaconate at 6 h (Extended Data Fig. 3h) and very weak inhibition (approximately 2% of inhibition by itaconate) by mesaconate and citraconate at 24 h. The much greater accumulation of succinate due to itaconate addition was also verified in two experiments using IAV-infected cells (Extended Data Fig. 3i,j).

### Citraconate is the strongest electrophile and SH-alkylator

**Extended Data Fig. 4:**
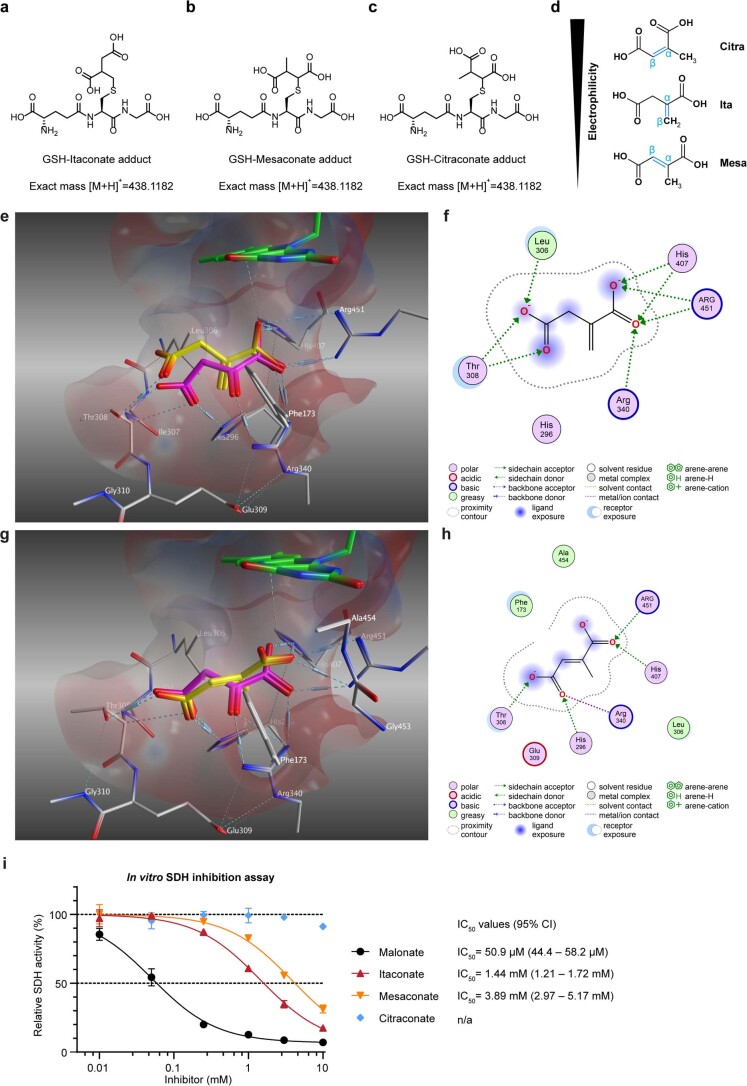
Citraconate is the most electrophilic isomer but the weakest SDH inhibitor. a–c, Structures of the GSH adducts of the itaconate isomers. d, Ranking of the three isomers in terms of electrophilicity. e–h, Molecular modeling prediction of non-covalent binding of itaconate and mesaconate to the active site of SDH. e, 3D binding mode of itaconate (yellow) compared to oxaloacetate (magenta) in the succinate binding site of human SDHA (PDB ID: 6VAX)^2^. Binding was attained entirely through a network of electrostatic attractions, that is, hydrogen bonds and salt bridges, (dashed lines) between the C1- and C4-carboxyl groups and the active site residues (Thr308, Arg340, His407, and Arg451). Electrostatic protein surface at the active site: positive (blue), negative (red), neutral (white). Flavin adenine dinucleotide (FAD, green). f, 2D ligand interactions of itaconate. g, Potential binding mode of mesaconate (yellow) compared to oxaloacetate (magenta). Similar to itaconate, the C1- and C4-carboxyl groups of mesaconate are the exclusive moieties responsible for binding through hydrogen bonds and ionic interactions (dashed lines). However, its rigid and planar structure could only establish partial contacts compared to itaconate. h, 2D ligand interactions of citraconate. i, Comparison of SDH inhibition by itaconate, mesaconate, and citraconate (*in vitro* assay using bovine mitochondria). SDH activity was measured in the presence of increasing concentrations of itaconate, mesaconate, citraconate and the control inhibitor malonate (*n*=3 independent assays, mean ±SD). Of the isomers, itaconate was the strongest SDH inhibitor, there was essentially no inhibition by citraconate.

It has been argued that itaconate inhibits SDH by covalently alkylating SH groups in the active site (SDHA)^17^. However, formation of Michael adducts with glutathione (Extended Data Fig. 4a–c) was actually most efficient with citraconate (which does not inhibit SDH), and was 8- and 48-fold higher compared with itaconate and mesaconate, respectively (Supplementary Fig. 2a–d and Supplementary Table 1). Indeed, lowest unoccupied molecular orbital and electrophilicity index (*ω*)^18^ calculations identified citraconate as the strongest, and mesaconate as the weakest electrophile (Extended Data Fig. 4d and Supplementary Table 1).

### Itaconate binds the active centre of SDH

Considering the discrepancy between the compounds’ electrophilicity (SH-alkylating ability) and SDH-inhibitory activity, we employed structural modelling to test alternate binding modes. Indeed, covalent binding to the nearest cysteine residues as the major mode of inhibition was deemed implausible for itaconate in both human and porcine SDHA (Supplementary Fig. 2e,f). Given the structural similarity of itaconate to succinate and substrate-like inhibitors such as oxaloacetate, itaconate may target the succinate-binding site via a competitive mechanism. Docking of the three isomers into the active site of human and porcine SDHA revealed that itaconate is predicted to bind in the succinate-binding site via electrostatic interactions with favourable binding energies similar to oxaloacetate (Extended Data Fig. 4e,f and Supplementary Fig. 2h). Mesaconate and citraconate show few contacts owing to the rigidity and planarity conferred by the extended conjugation. Noteworthy, mesaconate is an analogue of the succinate-oxidation product fumarate and it is plausible that it engages in interactions with SDHA (Extended Data Fig. 4g,h and Supplementary Fig. 2i), whereas the *cis*-configuration of citraconate seems to be unfavourable for binding (Supplementary Fig. 2j). An in vitro SDH activity assay using bovine mitochondria (whose active site is 100% conserved in human SDH) confirmed strong SDH inhibition by itaconate and essentially none by citraconate, but it did reveal a moderate degree of inhibition by mesaconate (Extended Data Fig. 4i). In agreement with recent biochemical evidence^19^ these results suggest that itaconate inhibits SDHA via direct non-covalent interactions with the active centre. The low degree of SDH inhibition by mesaconate in our cell-based experiments may be due to lower cytoplasmic–mitochondrial entry or because its strength of inhibition is insufficient to cause pronounced differences under steady-state conditions.

### Antiviral effects of the itaconate isomers

**Extended Data Fig. 5:**
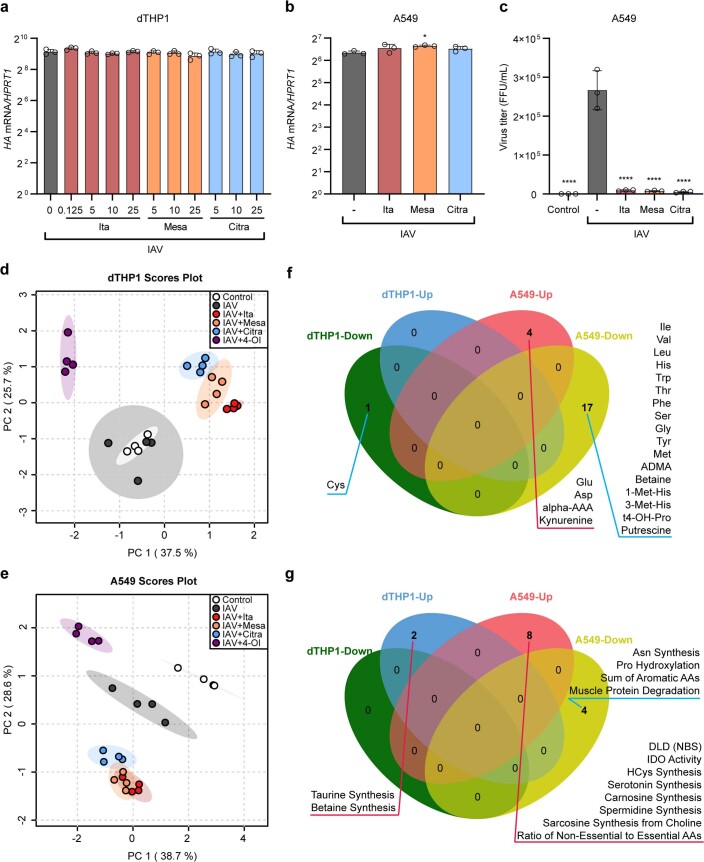
The itaconate isomers inhibit replication of influenza A virus (IAV) in the respiratory epithelial cell line A549. dTHP1 and A549 cells were left untreated or incubated with itaconate, mesaconate or citraconate (20 mM) or 4-octyl itaconate (4-OI, 125 µM), infected with IAV PR8M (MOI=1), and then incubated with fresh medium containing the treatments. Replication was measured in dTHP1 cells 12 h p.i. by expression of *HA* mRNA (RT-qPCR) and in A549 cells 24 h p.i. by measuring *HA* mRNA (RT-qPCR) and viral titers (foci forming assay). a, *HA* mRNA (dTHP1 cells). b, *HA* mRNA (A549 cells). c, IAV titers (A549 cells). *n*=4 biological replicates, means ±SD. One-way ANOVA followed by Dunnett’s multiple comparisons test. P values: * ≤0.05, ** ≤0.01, *** ≤0.001, **** ≤0.0001. Shared and unique effects of the itaconate isomers on amino acid metabolism in IAV infected dTHP1 and A549 cells. d-g, Same experimental set-up as a-c, except that 4-OI (125 µM) was used as additional treatment. Concentrations of 20 amino acids, 30 amino acid metabolites, and 9 biogenic amines were measured by HPLC-MS/MS (MxP Quant 500 kit, Biocrates) after 12 h for dTHP1, 24 h for A549 (*n*=4 per group). Additional data, including concentrations of individual analytes and values of metabolite indicators, are shown in Supplementary Fig. S3 and S4. d,e, PCAs based on the 59 amino acid-related analytes. d, In dTHP1 cells, infection leads to modest changes only, while the three isomers exert similar but clearly discernable effects, which differ profoundly from the impact of 4-OI. e, In A549 cells, infection exerts a much stronger impact on amino acid metabolism. f, Venn diagrams showing analytes that are differentially abundant (unpaired T-test, FC>1.3, FDR ≤0.05) in A549 and dTHP1 infected and uninfected cells. g, Venn diagrams for the metabolite indicators (66 sums and ratios of functionally related analytes, calculated with Biocrates MetaboIndicator^TM^ software). Source Data Table 1 contains the source data pertaining to d-g.

Itaconate derivatives are being studied as antiviral compounds and adjunct treatments to modify host responses in viral infections^9,10^. We therefore infected dTHP1 and A549 cells with IAV and treated them with non-toxic concentrations of the three isomers, as determined by 3-(4,5-dimethylthiazol-2-yl)-2,5-diphenyltetrazolium bromide (MTT) assay (Supplementary Fig. 1h). Analyses were performed at the time of maximal intracellular response to infection, that is, 12 h post infection (p.i.) (dTHP1) and 24 h p.i. (A549)^20^. Like primary human macrophages, dTHP1 cells support a non-productive IAV infection that features viral RNA replication and a strong intracellular response, but not the release of progeny virions. In this cell type, the treatments did not lead to a notable reduction in viral haemagglutinin (*HA*) messenger RNA (mRNA) synthesis (Extended Data Fig. 5a). By contrast, A549 cells support productive infection with the release of new virions. As expected, at 24 h p.i. there were high cellular levels of *HA* mRNA and high IAV titres in culture supernatants. Addition of the isomers did not affect *HA* mRNA levels, but, remarkably, all three strongly reduced viral titres in supernatants (itaconate, 30-fold; mesaconate, 36-fold; citraconate, 53-fold) (Extended Data Fig. 5b,c). Thus, all three isomers possess anti-IAV properties that apparently interfere with the production or release of new viral particles, presumably by interfering with a post-transcriptional process.

### Reprogramming of amino acid metabolism by the isomers

To search for metabolic correlates of their antiviral effects, and to obtain a broader view of the shared and unique effects of the three isomers on cell metabolism, we then employed this IAV infection model in a targeted analysis of amino acids and related metabolites. The impact of IAV on amino acid metabolism was weak in dTHP1 cells, but all three isomers effected pronounced alterations in amino acid metabolism in the infected cells that were distinct from the changes due to 4-OI (which was applied for comparison) and also appeared to differ among the isomers (Extended Data Fig. 5d). In A549 cells, the impact of infection was much stronger, but the isomers did effect further changes in amino acid-related metabolite populations (Extended Data Fig. 5e). The greater impact of infection on amino acid metabolism in A549 cells was evident in that 21 analytes were differentially abundant (17 of which were reduced), whereas only one analyte (Cys) was significantly changed in THP1 cells (Extended Data Fig. 5f). Likewise, infection changed the values of 12 metabolite indicators in A549 cells, but only two in dTHP1 cells (Extended Data Fig. 5g). Treatment of IAV-infected dTHP1 cells with the isomers resulted in 24 significantly changed analytes, 19 of which were decreased (Supplementary Fig. 3a,b). Some effects were uniquely associated with one isomer, but others were shared by two or all three. For instance, all three isomers increased predicted prolyl hydroxylase activity (an enzymatic activity important for hypoxia-inducible factor 1α [HIF-1α] destabilization), but itaconate and citraconate had the greatest effects on proline and trans-4-hydroxyproline levels (Supplementary Fig. 4j–l). Mesaconate effected the highest number of significant changes; in particular, it broadly reduced amino acid levels, which was evident in all amino acid subclasses (Supplementary Figs. 3c and 4b,c). In A549 cells, the treatments led to fewer significant changes, probably because of the stronger impact of the infection (Supplementary Fig. 3e–h). This is exemplified by Arg, His, Lys and Trp, four amino acids that are important for influenza virus replication and are typically reduced in productive influenza virus infection^21^. All of these were reduced significantly in infected A549 cells, but (as opposed to dTHP1 cells) the treatments did not lead to a notable further reduction (Supplementary Fig. 4d–g). Nonetheless, distinct and shared effects of the isomers were evident in A549 cells. For instance, all three isomers increased levels of kynurenine (Kyn) and predicted activity of IDO1, a potentially immunosuppressive enzyme responsible for the conversion of Trp to Kyn and generation of NAD^+^ as the end-product of the Trp–Kyn–NAD^+^ pathway (Supplementary Fig. 4h,i), whereas itaconate preferentially increased Thr, Ala, Ser, Asp and asymmetric dimethylarginine levels (Supplementary Fig. 4m–q). Polyamines play important roles in virus–host interactions at multiple, mostly post-transcriptional, steps^22^. The three measured polyamines were intermediates of the spermidine/spermine N1-acetyltransferase pathway, and their depletion can inhibit RNA virus replication^23^. Indeed, all three isomers tended to decrease polyamine levels in infected A549 cells, although significance at *P* < 0.05 was achieved only in the case of the precursor ornithine (Supplementary Fig. 4t–y). Taken together, these results demonstrate that supraphysiological concentrations (mimicking pharmacological application) of the itaconate isomers exert profound effects on amino acid metabolism in IAV-infected cells, which can differ substantially according to isomer, cell type and type of infection (productive versus non-productive), and may functionally relate to antiviral networks such as the polyamine pathway.

### Citraconate is the strongest NRF2-activating isomer

**Fig. 2:**
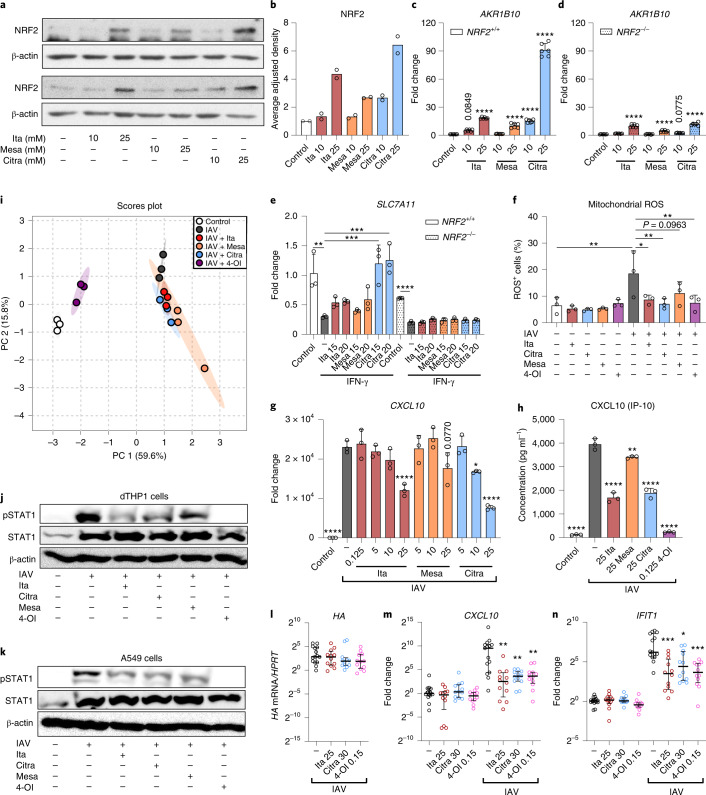
Differential effects of the isomers on NRF2 stabilization, mitochondrial ROS levels and IFN-signaling. **a**–**d**, Citraconate is the strongest NRF2 agonist. **a**,**b**, Stabilization of NRF2 protein in wild-type cells. Western blot (3 h after stimulation) of two independent experiments (**a**) and combined densitometric measurements of the bands at 110–120 kDa corresponding to NRF2 (**b**) (*n* = 2). **c**,**d**, Expression of the NRF2-inducible *AKR1B10* mRNA in wild-type and *NRF2*^*–/–*^ cells 16 h after stimulation. Cells were pre-treated with itaconate isomers for 6 h or left untreated and then stimulated with IFN-γ (300 U ml^−1^) for 10 h in the presence or absence of the isomers (*n* = 6, mean ± s.d.). **e**, Citraconate rescues *SLC7A11* mRNA suppression by IFN-γ in wild-type but not *NRF2*^*–/–*^ HaCaT cells (RT–qPCR). **f**, Citraconate exerts anti-oxidative effects similar to itaconate. dTHP1 cells were infected with IAV and ROS were measured at 12 h p.i. (itaconate isomers = 25 mM, 4-OI = 125 µM). **g**–**n**, Shared and distinct immunomodulatory effects of the isomers and 4-OI. dTHP1 cells were infected with IAV in the absence or presence of itaconate, mesaconate, citraconate (variable concentrations) or 4-OI (125 µM), and inflammation-related molecules were measured at 12 h p.i. **g**,**h**, Levels of *CXCL10* mRNA in cell pellets and CXCL10 protein in supernatant. **i**, Differential effects on inflammation-related polypeptides in cell culture supernatants (27-plex assay; separate experiment from **g**,**h**; isomer concentrations = 20 mM, 4-OI = 125 µM.) PCA based on concentrations of 25 targets that passed quality assessment. **c**–**i**, *n* = 3 biological replicates, mean ± s.d. **j**,**k**, Itaconate isomers reduce STAT1 phosphorylation. A549 and dTHP1 cells (one representative blot of two replicas) were pretreated with itaconate isomers (25 mM) or 4-OI, infected with IAV for 2 h and treated for 10 h (dTHP1) and 22 h (A549) (anti-P-STAT1 immunoblot). **l**–**n**, Citraconate reduces IAV-induced IFN responses in human lung tissue. Lung explants were infected with IAV (2 × 10^5^ FFU ml^−1^) in the presence or absence of itaconate, citraconate and 4-OI at the indicated concentrations (mM). Expression of target mRNAs was measured at 24 h p.i. (five explants, three pieces per explant, total *n* = 15, median, interquartile range). Citra, citraconic acid; Ita, itaconic acid; Mesa, mesaconic acid. *P ≤ 0.05, **P ≤ 0.01, ***P ≤ 0.001, ****P ≤ 0.0001; one-way ANOVA with Dunnett’s multiple comparisons test, except **l**–**n** (two-tailed Mann–Whitney *U* test with Bonferroni correction). Source data

**Extended Data Fig. 6:**
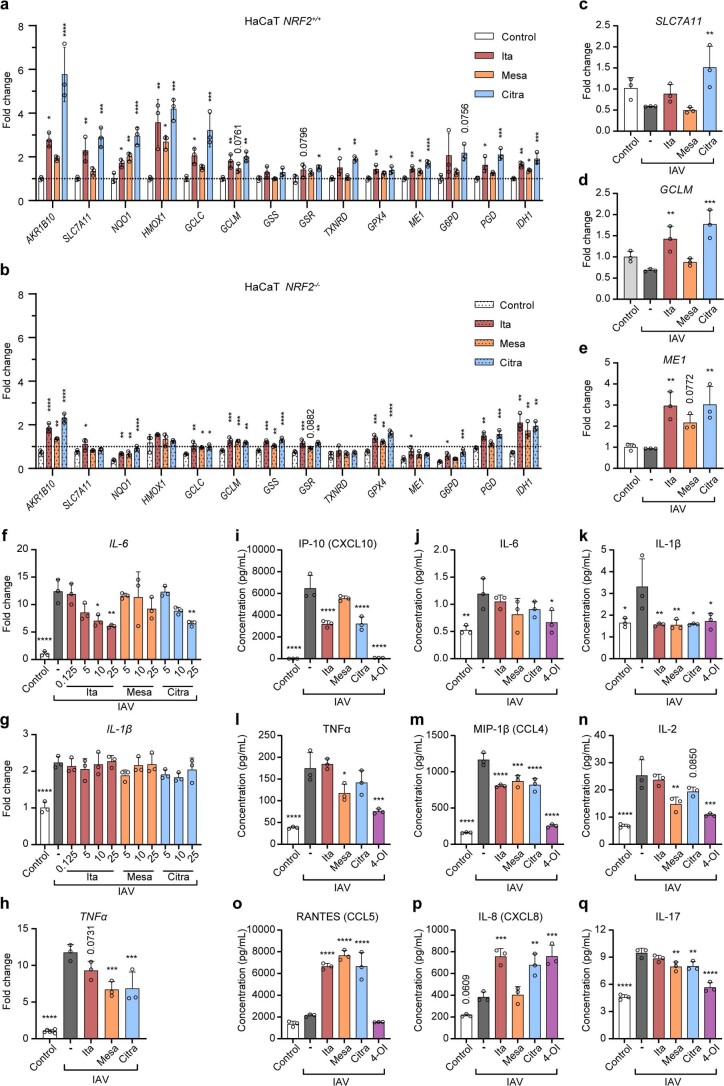
Citraconate is the strongest NRF2 agonist. a,b, Expression of 14 potentially NRF2-inducible genes in WT and *NRF2*^*–/–*^ HaCaT cells 17.5 h after administration of itaconate isomers (20 mM). The greatest difference between expression in WT and KO cells is seen upon administration of citraconate. c–e, Induction of *SLC7A11*, *GCLM*, and *ME1* mRNA in IAV-infected dTHP1 cells by citraconate but not mesaconate. Cells were pretreated with itaconate isomers (20 mM) for 12 h, incubated with virus-containing medium for 2 h (MOI=1), and then incubated with fresh medium containing itaconate isomers for a further 10 h. f–q, Immunomodulatory effects of the itaconate isomers. IAV infection experiment identical to c–e. f–h, Expression of the indicated mRNAs in cells (RT-qPCR). i–q, Concentrations of the indicated cytokines/chemokines in culture supernatants (multiplex microbead assay). Source Data Table S2 contains the raw data pertaining to i–q. a-q, *n*=3 biological replicates, means ±SD. P values: * ≤0.05, ** ≤0.01, *** ≤0.001, **** ≤0.0001; one-way ANOVA with Dunnett’s multiple comparisons test.

Considering that citraconate is the strongest electrophile of the three isomers, we then compared the ability of the isomers to activate the KEAP1–NRF2 signalling pathway^24^. Citraconate exerted the strongest stabilizing effect on NRF2 in HaCaT keratinocytes and induced mRNA encoding aldo-keto reductase family 1 member B10 (AKR1B10, a downstream factor induced by NRF2) most strongly (Fig. 2a–c). In *NRF2*^*–/–*^ HaCaT cells, *AKR1B10* mRNA expression was lower at baseline and there was significant induction only at the higher citraconate concentrations, with a lower fold change than in wild-type cells (Fig. 2d). When assaying 14 potentially NRF2-regulated genes^25^, citraconate effected the, overall, strongest induction in wild-type cells, whereas expression of most targets at baseline and after stimulation was lower in *NRF2*^*–/–*^ cells (Extended Data Fig. 6a,b). IFN-γ stimulation of wild-type HaCaT cells resulted in marked downregulation of *SLC7A11* mRNA, which was rescued by citraconate in the wild-type but not the *NRF2*^*–/–*^ cells (Fig. 2e). Similarly, in IAV-infected dTPH1 cells application of citraconate increased expression of *SLC7A11*, *GCLM* and *ME1* mRNA, whereas mesaconate had no effect (Extended Data Fig. 6c–e). These inducing effects were modest, which agreed with the classification of the isomers as moderate electrophiles (Supplementary Table 1). Itaconate can reduce reactive oxygen species (ROS) levels^14^. We compared anti-ROS activity of the three isomers in IAV-infected dTHP1 cells. Infection led to a significant increase in the number of mitochondrial ROS-positive cells, which was reversed significantly by itaconate and citraconate, whereas ROS reduction by mesaconate was only marginally significant (Fig. 2f).

### Immunomodulatory effects of the isomers

Itaconate has strong immunomodulatory properties^3,4^, and we thus tested whether mesaconate and citraconate also modify inflammatory responses. In IAV-infected dTHP1 cells, all three isomers reduced mRNA encoding C-X-C motif chemokine ligand 10 (CXCL10), whereas reduction of CXCL10 protein in cell supernatants was greater by itaconate and citraconate (Fig. 2g,h). Itaconate and citraconate reduced interleukin-6 (*IL-6*) mRNA, none affected *IL-1β* transcription, and only mesaconate and citraconate reduced tumour necrosis factor-α (*TNFα*) mRNA (Extended Data Fig. 6f–h). In an independent experiment, IAV infection led to substantial reprogramming of cytokine/chemokine populations in supernatants, which was strongly modulated by addition of 4-OI, but less by the three isomers (Fig. 2i). Nonetheless, common and unique effects of the three isomers were seen (Extended Data Fig. 6i–q). Again, only itaconate and citraconate reduced CXCL10 protein levels significantly. All three isomers reduced IL-1β and macrophage inflammatory protein-1β (CCL4) concentrations, whereas only mesaconate reduced IL-2 and tumour necrosis factor-α. Of note, all three isomers increased concentrations of the chemokine CCL5 (RANTES), whereas only itaconate and citraconate markedly increased IL-8 concentrations. This analysis revealed that: (1) 4-OI exerted the strongest ‘normalizing’ effects on cytokine/chemokine release from infected dTHP1 cells; (2) exogenously applied mesaconate and citraconate possess common and unique immunomodulatory properties (some of which are shared with itaconate); and (3) the potential functional consequences may relate in part to differences in recruitment of additional inflammatory cells. To test whether the observed reduction in *CXCL10* mRNA and protein levels was due to diminished canonical type I IFN signalling, we assessed the effects of the isomers on signal transducer and activator of transcription 1 (STAT1) phosphorylation in dTHP1 and A549 cells. IAV infection greatly increased levels of unphosphorylated and phosphorylated STAT1, and all three isomers reduced levels of phosphorylated but not unphosphorylated STAT1 (Fig. 2j,k). Mesaconate had a somewhat weaker effect on dTHP1 cells, whereas 4-OI was by far the most potent in both cell types. The anti-IFN potential of citraconate was also verified in ex vivo cultured human lung tissue. IAV infection led to the expected increase in viral *HA*, *IFIT1* and *CXCL10* mRNA. Although there was no significant reduction in viral *HA* RNA levels, citraconate significantly reduced both *IFIT1* and *CXCL10* expression (Fig. 2l–n).

### Citraconate is a direct inhibitor of ACOD1 catalysis

**Fig. 3:**
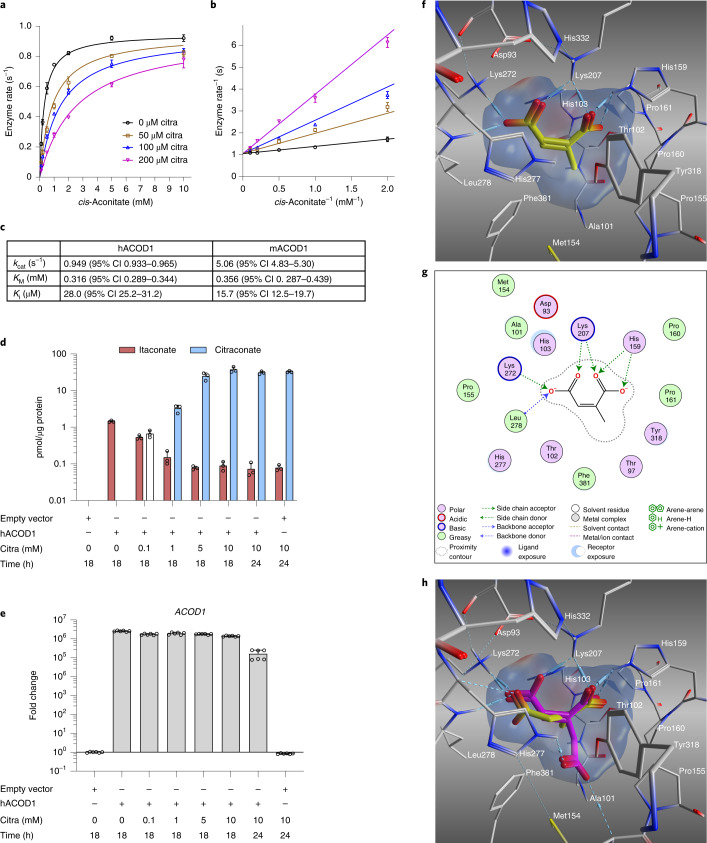
Citraconate is a competitive inhibitor of ACOD1 that binds in the substrate-binding site. **a**–**c**, Cell-free assay. Recombinant hACOD1 was incubated with increasing concentrations of substrate (*cis*-aconitate) and inhibitor (citraconate) and itaconate accumulation was measured by HPLC. *n* = 3 independent assays, mean ± s.d. The line represents a curve fit to the Michaelis–Menten equation with a competitive inhibitor. **b**, Lineweaver–Burk plot of the data shown in **a** (mean ± s.d.). **c**, Summary of enzyme and inhibition kinetics based on **a** (hACOD1) and data shown in Extended Data Fig. 7a,b (mACOD1). **d**,**e**, Cell-based assay. A549 cells were transfected with a plasmid overexpressing hACOD1 and incubated with increasing concentrations of citraconate. Intracellular citraconate and itaconate concentrations were measured by HPLC–MS/MS. Adding citraconate leads to reduced itaconate accumulation in a dose-dependent manner (**d**), which is not due to decreased *hACOD1* transcription (**e**). *n* = 3 biological replicates (**d**), *n* = 6 biological replicates (**e**), mean ± s.d. **f**–**g**, Putative binding mode of citraconate (yellow) in the active site of hACOD1 (PDB ID: 6R6U)^2^. **f**, The C1- and C4-carboxyl groups of citraconate are optimally anchored in the active site through a network of electrostatic attractions; that is, hydrogen bonds and salt bridges (dashed lines) with the residues His159, Lys207, Lys272 and Leu278. Electrostatic protein surface at the active site: positive (blue), negative (red) and neutral (white). **g**, Two-dimensional ligand interactions. **h**, Molecular modelling of itaconate (yellow) compared with *cis*-aconitate (magenta) revealing fewer interactions between the two carboxyl groups of itaconate and the basic residues of the hACOD1 active site mainly due to its non-planar and flexible structure. CI, confidence intervals; citra, citraconate.

**Extended Data Fig. 7:**
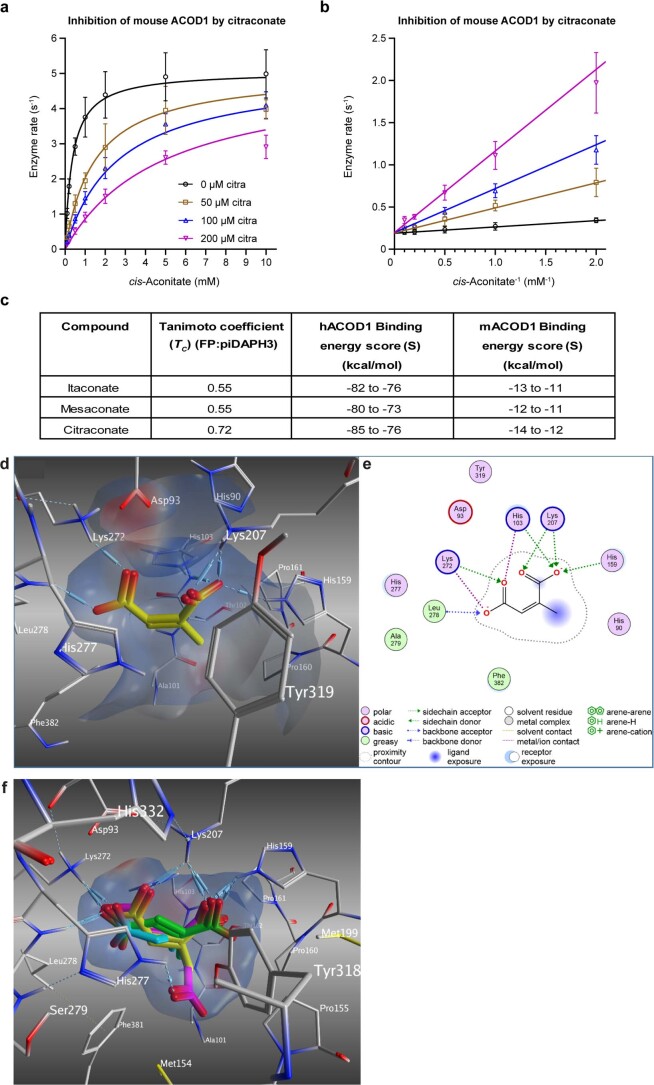
Citraconate is a competitive inhibitor of ACOD1. a,b, Cell-free assay. Recombinant mACOD1 was incubated with increasing concentrations of substrate (*cis*-aconitate) and inhibitor (citraconate) and itaconate accumulation was measured by HPLC. *n*=3 independent assays, means ±SD. Kinetic data pertaining to the values listed in Fig. 3c. The line represents a curve fit to the Michaelis–Menten equation with a competitive inhibitor. b, Lineweaver–Burk plot of the data shown in a. c, Similarity to *cis*-aconitate (Tanimoto coefficient) and binding energies of the itaconate isomers to human and murine ACOD1. d, Putative binding mode of citraconate (yellow) in the active site of mouse ACOD1 (PDB ID: 6R6T)^1^. The C1- and C4-carboxyl groups of citraconate bind electrostatically via hydrogen bonds and ion contacts (dashed lines) to the active site residues (His103, His159, Lys207, Lys272, and Leu278). Electrostatic protein surface at the active site: positive (blue), negative (red), neutral (white). e, 2D ligand interactions of citraconate. f, Overlay of putative binding modes of citraconate (yellow), itaconate (cyan), and mesaconate (green) compared to that of the substrate *cis*-aconitate (magenta) in the active site of human ACOD1 (PDB ID: 6R6U)^1^. Only citraconate can adopt the same binding mode as *cis*-aconitate, where the *cis*-oriented carboxyl groups are fully involved in the interactions with the active-site residues (His159, Lys207, Lys272, and Leu278). In contrast, the carboxyl groups of itaconate and mesaconate interact partially resulting in lower binding affinity.

Of several compounds with similarity to *cis*-aconitate, only citraconate proved to be an ACOD1 inhibitor. Remarkably, it did not affect activity of *Aspergillus* ACOD1, but 10 mM citraconate decreased the activity of human and murine ACOD1 by about 90% (Fig. 3a–c and Extended Data Fig. 7a,b). ACOD1 inhibition by citraconate was also verified in ACOD1-expressing A549 cells. Indeed, there was a dose-dependent decrease in itaconate accumulation in the presence of citraconate (Fig. 3d). This was not due to decreased expression of *ACOD1*, because mRNA levels were unaffected (Fig. 3e). The concentration of citraconate in the medium that resulted in half-maximal inhibition of ACOD1 activity (IC_50_) was found to be 50 µM (Supplementary Fig. 5d,e). Comparing the pharmacophore fingerprint of the three itaconate isomers with that of *cis*-aconitate showed that citraconate has the highest Tanimoto coefficient, that is the greatest similarity to *cis*-aconitate (Extended Data Fig. 7c). This suggested that it binds the active site of ACOD1 and acts as a substrate analogue. Indeed, structural modelling predicted that it binds favourably into the active site of human ACOD1^2^ and the murine homologue^2^ in a mode similar to *cis*-aconitate (Fig. 3f–h and Extended Data Fig. 7d,e), whereas the non-planar itaconate and the *trans*-isomer mesaconate display lower binding energies and fit less optimally (Extended Data Fig. 7f).

**Fig. 4:**
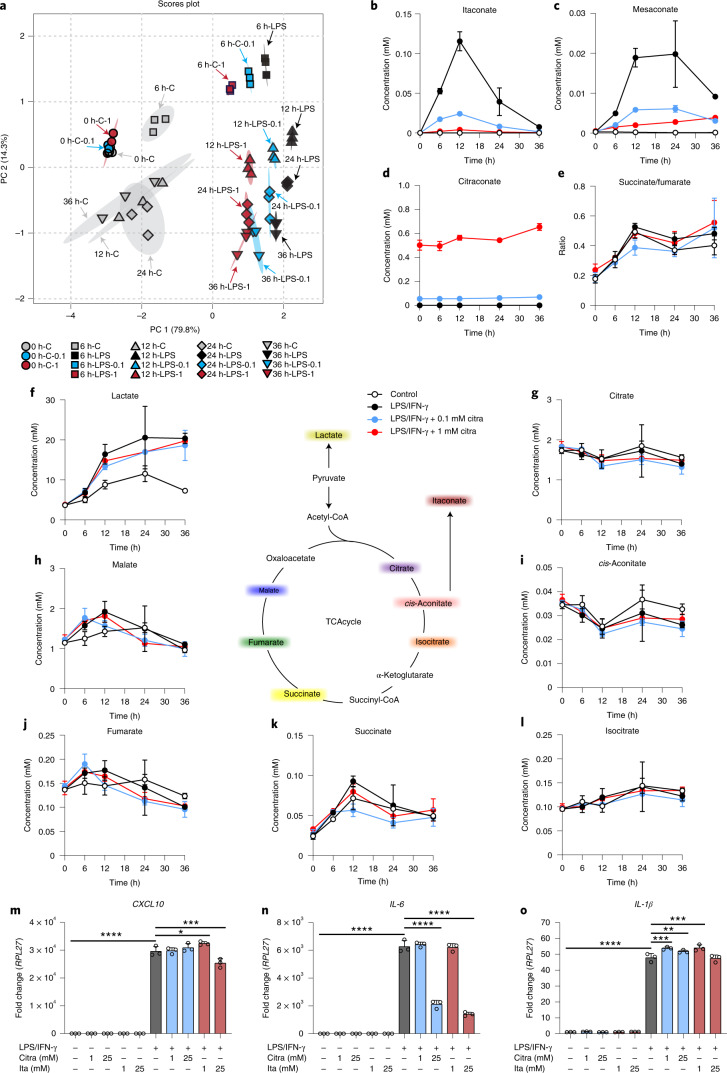
Citraconate prevents itaconate and mesaconate accumulation in LPS/IFN-γ-mediated macrophage activation. dTHP1 cells were stimulated with LPS (200 ng ml^−1^) and IFN-γ (400 U ml^−1^) for the indicated times in the absence or presence of 0.1, 1, 5, 10 and 25 mM citraconate. Unstimulated dTHP1 cells treated for 6 h were used as an additional control. Itaconate isomers, lactate and the same TCA intermediates as in Fig. 1 were measured by LC–MS/MS. Concentrations 0.1 and 1 mM citraconate in medium resulted in strong ACOD1 inhibition at physiologically plausible intracellular citraconate concentrations and were thus used for the analyses shown in **a**–**l**. Data obtained with the 5–25 mM concentrations were additionally used to calculate IC_50_ values. **a**, PCA based on all analytes except citraconate. **b**–**e**, Time course of itaconate (**b**), mesaconate (**c**), citraconate (**d**) concentrations and succinate/fumarate ratio (**e**). **f**–**l**, Concentrations of lactate and selected TCA intermediates. **m**–**o**, ACOD1 inhibition by citraconate does not have a strong effect on inflammation in dTHP1 cells activated by LPS-IFN-γ. Experimental set-up identical to **a**–**l**. Levels of the indicated mRNAs were measured by RT–qPCR 12 h after stimulation. **m**, *CXCL10* mRNA. **n**, *IL-6* mRNA. **o**, *IL-1β* mRNA. Citra, citraconic acid; ita, itaconic acid. **b**–**o**, *n* = 3 biological replicates, mean ± s.d. *P ≤ 0.05, **P ≤ 0.01, ***P ≤ 0.001, ****P ≤ 0.0001; one-way ANOVA with Dunnett’s multiple comparisons test.

To study the cellular kinetics of the effects of ACOD1 inhibition in a classic model of macrophage activation, we performed a 36 h time-course experiment of LPS/IFN-γ activation of dTHP1 cells in the presence of two different concentrations of citraconate and measured concentrations of TCA intermediates, itaconate and mesaconate. PCA demonstrated relatively mild effects of a 6 h pretreatment with citraconate on unstimulated cells, but progressive TCA reprogramming during LPS/IFN-γ stimulation (Fig. 4a). Of note, citraconate treatments tended to reverse the changes induced by LPS/IFN-γ, which was mostly (but not exclusively) due to prevention of itaconate and mesaconate accumulation. Specifically, in untreated stimulated cells, itaconate accumulated rapidly and peaked at 12 h, whereas 1 mM citraconate essentially prevented itaconate and greatly reduced mesaconate accumulation (Fig. 4b,c). The inhibitory effect of citraconate persisted over 36 h, which agreed with the steady levels of intracellular citraconate throughout the time course (Fig. 4d). Unexpectedly, lack of itaconate synthesis was not accompanied by an increase in *cis*-aconitate levels at early time points, which might be due to the decreased availability of its precursor citrate (Fig. 4g,i). Both citraconate concentrations reduced lactate levels; in particular, the 0.1 mM concentration led to a moderate decrease in succinate levels and succinate/fumarate ratio, suggesting a modest increase in SDH activity due to relief of SDH inhibition by endogenous itaconate. The measured IC_50_ value of intracellular citraconate for ACOD1 catalysis (Supplementary Fig. 5a–c) agreed well with values measured in the cell-free assay (Fig. 3c).

**Extended Data Fig. 8:**
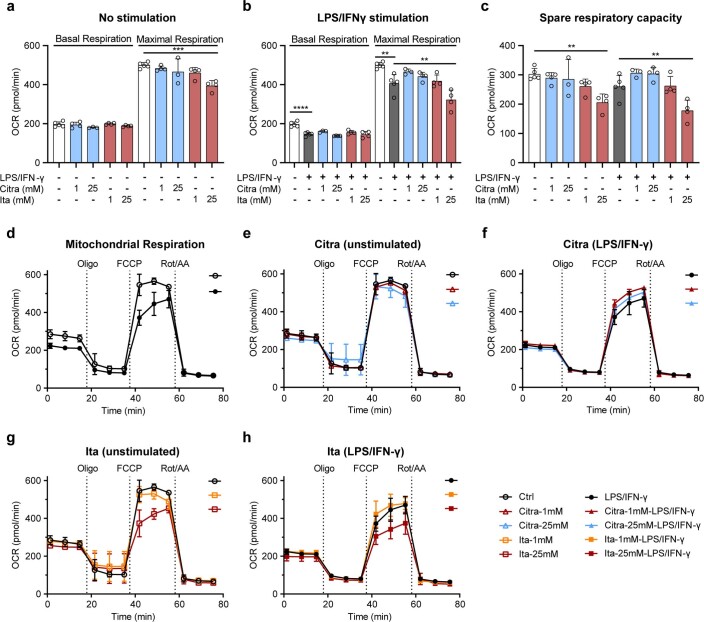
Itaconate, but not citraconate, depresses mitochondrial respiration in LPS/IFNγ-activated dTHP1 cells. LPS/IFNγ stimulation and treatment with citraconate or itaconate (1 mM or 25 mM) were performed as in Fig. 4m-o. Mitochondrial respiration was measured by Seahorse assay in unstimulated dTHP1 cells or after 12 h of stimulation. a-c, Bar graphs showing oxygen consumption rate (OCR) due to basal respiration, maximal respiration (a and b), and spare respiratory capacity (c). 25 mM itaconate significantly reduced maximal respiration and spare respiratory capacity in LPS/IFNγ-induced dTHP1 cells, whereas there was a tendency (p=0.076 and 0.096 respectively) of 1 mM citraconate to prevent this decline, and there was a tendency of 25 mM itaconate to normalize spare respiratory capacity (p=0.082). In a *n* (biological replicates) were as follows: untreated = 5; 1 mM Citra = 4; 25 mM Citra = 3; 1 mM Ita = 4; 25 mM Ita = 4. In b *n* were as follows: untreated = 5; 1 mM Citra = 5; 25 mM Citra = 3; 1 mM Ita = 4; 25 mM Ita = 4. Results in c are computed from the experiment shown in a and b and therefore have the same *n* as the respective treatments in a and b. Means ±SD; one-way ANOVA followed by Dunnett’s multiple comparisons test. P values: * ≤0.05, ** ≤0.01, *** ≤0.001, **** ≤0.0001. d-h, OCR output curves that form the basis of the graphs shown in a-c. d, Unstimulated vs. LPS/IFNγ-stimulated cells. e, Citraconate treatment, unstimulated cells. f, Citraconate treatment, LPS/IFNγ-stimulated cells. g, Itaconate treatment, unstimulated cells. h, Itaconate treatment, LPS/IFNγ-stimulated cells. Results in d-h are computed from the experiment shown in a and b and therefore have the same *n* as the respective treatments in a and b. Means ±SD. P values: * ≤0.05, ** ≤0.01, *** ≤0.001, **** ≤0.0001; one-way ANOVA with Dunnett’s multiple comparisons test. Abbreviations: FCCP = carbonyl cyanide-4 (trifluoromethoxy) phenylhydrazone; Oligo = oligomycin; Rot = rotenone; AA = Antimycin A.

Considering that ACOD1 inhibition resulted from substantially lower citraconate concentrations than the immunomodulatory effects (0.03–1 mM versus 10–25 mM), we then tested whether the lower concentration would result in an inflammation-related phenotype, which would probably be due to pharmacologic abrogation of endogenous itaconate synthesis. To this end, we activated dTHP1 cells with LPS/IFN-γ, which results in maximal activation and much higher ACOD1 expression than infection with viruses such as IAV. Although LPS/IFN-γ stimulation did result in vigorous upregulation of *IL-1β, IL-6* and *CXCL10* mRNA, treatment with 1 mM citraconate or 1 mM itaconate (used as a control that does not inhibit ACOD1) did not affect *CXCL10* and *IL-1β* mRNA to a noteworthy extent. By contrast, the 25 mM concentration of either isomer reduced *IL-6* mRNA levels (Fig. 4m–o). Thus, pharmacologic inhibition of endogenous itaconic acid synthesis by low-dose citraconate did not have a major impact on the inflammatory phenotype of maximally activated dTHP1 cells, whereas a reduction in *IL-6* mRNA was observed at the higher (immunomodulatory) concentration. In a separate experiment, we then tested whether low/high concentrations of the two isomers would affect mitochondrial respiration. Itaconate at 25 mM, but not citraconate, reduced maximal respiration and spare capacity of unstimulated dTHP1 cells or dTHP1 cells stimulated with LPS/IFN-γ (Extended Data Fig. 8), which was consistent with reduced flux through the electron transport chain due to SDH inhibition and agreed well with our observation that citraconate does not inhibit SDH (Extended Data Fig. 4). LPS/IFN-γ stimulation reduced basal respiration, maximal respiration and spare respiratory capacity, and 1 mM citraconate tended to normalize maximal respiration and spare respiratory capacity, whereas 25 mM citraconate tended to normalize only spare respiratory capacity. The effects of low-dose citraconate could be explained by a model in which ACOD1 inhibition leads to reduced endogenous itaconate accumulation, which in turn increases electron flux by relieving itaconate-mediated SDH inhibition.

## Discussion

Taken together, our results reveal mesaconate and citraconate as previously underappreciated close relatives of itaconate that exert biologically relevant unique and shared effects on metabolism, inflammation and virus infection. The Graphical Abstract and Extended Data Table 1 summarize and compare key features of the three isomers. The observed differences in effects are probably due to differences in electrostatic interactions, steric properties and electrophilicity, which in turn could be affected by nucleophilicity of potentially partnering Michael donors. The marked disparities among the three isomers in the inhibition of ACOD1 (citraconate) or SDH (itaconate) exemplify the effects of electrostatic and steric differences, and the greater ability of citraconate to induce NRF2 is probably due to its stronger electrophilicity. The latter may also explain its greater effect on IAV production from A549 cells, because inhibition via the exportin 1 pathway by compounds based on an itaconate backbone has been shown to inhibit export of viral ribonucleoprotein into the cytoplasm by SH-alkylation of a critical Cys residue^9^. Citraconate may, therefore, effect the strongest reduction in IAV replication in A549 cells because of its greater ability to alkylate this residue. It has been proposed that itaconate exerts antiviral effects by virtue of inhibiting SDH^26^, but SDH inhibition is unlikely to play a role in the anti-influenza effects of the three isomers, because citraconate (which does not inhibit SDH) inhibited IAV replication the most efficiently.

We identified citraconate as the first endogenous ACOD1 inhibitor, but the physiological consequences at the organismal level remain unknown. In healthy mice, we previously found the highest citraconate levels in lymph nodes^5^. Although the cell type(s) harbouring citraconate remain unknown, it is tempting to speculate that it may modulate immune processes in lymph nodes by inhibiting ACOD1. Because there is no evidence of citraconate occurring in myeloid cells, such ACOD1 inhibition would probably need to occur in a paracrine manner. Its role as an endogenous NRF2 agonist also requires further exploration, particularly because the cell types harbouring citraconate in vivo are not known. Considering that citraconate was not detected in activated dTHP1 cells, we consider it unlikely that it plays a more prominent role in NRF2 signalling in macrophages than itaconate. Our results substantiate a previously formulated model that mesaconate is derived from itaconate^6,7^ (also see the accompanying paper by He et al.^27^), but one can only speculate about the physiological origin(s) of citraconate in humans. We show that it is not derived from itaconate or mesaconate. Based on studies of patients with methylmalonic aciduria, it has been proposed that it is a derivative of the BCAA isoleucine by way of tiglyl-CoA^8^. BCAA metabolism is a critical component of energy and immune homeostasis, and citraconate may thus constitute part of the regulatory networks governing BCAA metabolism. Overall, citraconate appears to be the isomer with the greater potential for translational drug development. Its multifaceted features such as anti-inflammatory, anti-oxidative and antiviral properties make it a particularly attractive backbone for the development of pharmacologically optimized drugs to treat disorders driven by inflammation, viral infection or both. The pharmacological relevance of ACOD1 inhibition by citraconate remains to be explored in vivo. Our studies with low-dose (1 mM) citraconate suggest that abrogating endogenous itaconate synthesis may not have a strong impact on maximal activation of human macrophages, at least with respect to *CXCL10*, *IL-6* and *IL-1β* mRNA. Most work on the role of endogenous itaconate has been performed in mice, and both hyper- and hypo-inflammatory phenotypes of bone marrow-derived macrophages from *ACOD1*^−/−^ mice have been described^28,29^. Moreover, murine ACOD1 is much more active than the human enzyme, resulting in five- to tenfold higher itaconate levels in activated macrophages compared with humans^1,2^. It is thus possible that ACOD1 plays a less-prominent role in regulating inflammation in humans than in mice. In addition, the consequences of ACOD1 inhibition may become fully manifest only at the organism level and may not be apparent in cellular models. Indeed, we recently found that *CXCL10* expression in IAV-infected bone marrow-derived macrophages did not differ between wild-type and *ACOD1*^*–/–*^ mice, whereas histologically assessed pulmonary inflammation was significantly higher in *ACOD1*^*–/–*^ mice^10^. In contrast to inflammation, low-dose citraconate did have a marked effect on TCA cycle intermediates and tended to improve mitochondrial respiration in dTHP1 cells stimulated with LPS/IFN-γ. Intriguingly, itaconate accumulation has been shown to correlate with immune paralysis, as found for example in sepsis^30^. Citraconate derivatives may thus prove beneficial in the treatment of patients with end-stage sepsis or other scenarios characterized by exhaustion of innate immunity. In addition, considering the protumorigenic effects of ACOD1, citraconate may prove useful as a scaffold for the development of ACOD1 inhibitors for cancer therapy.

Our results regarding mesaconate complement those reported in the accompanying paper by He et al.^27^. Using stable isotope-assisted metabolic flux analysis, these authors demonstrate that mesaconate is indeed a catabolite of itaconate. They report immunomodulatory effects of mesaconate in murine macrophages, which appear to be largely Nrf2-independent, and they show beneficial effects of mesaconate in a mouse model of LPS-induced sepsis. In addition, they show that itaconate is a much stronger SDH inhibitor than mesaconate. However, their results differ from ours in that they do see some SDH inhibition by mesaconate when analysing whole cells. The differences between the observations by He et al. and by us may be due to differences in the species and assays used. Nonetheless, taken together the results from both papers suggest that SDH inhibition by mesaconate is unlikely to have a strong biological impact.

## Methods

### In vitro ACOD1 activity assay

hACOD1 (amino acids 4–461) and mACOD1 (amino acids 4–462) were produced in *Escherichia coli*, purified as described previously^2^ and stored in GF buffer (10 mM HEPES, pH 7.4, 10% v/v glycerol, 150 mM NaCl). Assays were performed in triplicate. Enzymes in 46.6 µl of GF buffer (30 µg mACOD1 or 90 µg hACOD1) or 46.6 µl of GF buffer without enzyme were mixed with 3.3 µl of 15 or 150 mM *cis*-aconitate (pH 6.5), resulting in final *cis*-aconitate concentrations of 1 or 10 mM. The assays (50 μL) were incubated at 37 °C for 2 h and then placed on ice. For extraction of organic acids, 800 µl of extraction solvent (acetonitrile/methanol 1:1) was then added to the reaction and the resulting volume was transfered to a fresh tube. The now empty reaction tubes were rinsed with water (150 µl), which was subsequently added to the tubes containing activity assay in extraction solvent, resulting in a final volume of 1000 µl. Samples were mixed for 30 s and stored at –80 °C.

### ACOD1 inhibitor screen

A set of carboxylic acids (pyruvic acid, succinic acid, citric acid, citraconic acid, (2*E*)-2-ethylbut-2-enedioic acid (EN300-234639, Enamine), dl-isocitric acid and *trans*-aconitic acid) were screened as potential inhibitors. The acids were dissolved in water and neutralized. Enzyme assays were performed in sodium phosphate buffer, pH 7.4, with 2 mM *cis*-aconitate and 10 mM of the potential inhibitor. The amount of enzyme that would decarboxylate approximately half of the substrate was used per 150-µl assay (20 µg of *Aspergillus* ACOD1, amino acids 12–490, 24 µg of hACOD1 and 5 µg of mACOD1). Itaconate was quantified using high-performance liquid chromatography (HPLC), as described below.

### Quantification of inhibition of ACOD1 enzyme activity by citraconate (cell-free assay)

Human, mouse and *Aspergillus* ACOD1 were prepared as described previously^2^ and stored in GF buffer (10 mM HEPES, pH 7.4, 10% v/v glycerol, 150 mM NaCl). Solutions of the inhibitor (citraconic acid; Acros) and the substrate (*cis*-aconitic acid; Sigma-Aldrich), both pH 6.5 (NaOH), were stored at –20 °C. For the assay, 125 μl of 0.2 M sodium phosphate buffer, pH 6.5, was mixed on ice with 5 μl of enzyme, 10 µl of citraconate (or 10 µl of water) and 10 μl of *cis*-aconitate. The final concentrations of the inhibitor were 50, 100 and 200 µM. The following combinations of enzyme amount and substrate concentration were used: 2 µg of hACOD1 or 0.4 µg of mACOD1 with 0.1, 0.2 or 0.5 mM substrate; 3 µg of hACOD1 or 0.6 µg of mACOD1 with 1 or 2 mM substrate; 5 µg of hACOD1 or 1 µg of mACOD1 with 5 or 10 mM substrate. Assays were done in triplicate. Incubation at 37 °C for 10 min was immediately followed by heat inactivation of the enzyme at 95 °C for 3 min. The protein precipitate was pelleted by centrifugation for 1 h. Supernatants were acidified with 100 μl of 100 mM H_3_PO_4_. Itaconic acid was measured by HPLC (Shodex RSpak DE-413 column, 1 ml min^−1^ 10 mM H_3_PO_4_, injection volume 10–20 µl, detection at 210 nm). The resulting curves were fitted using GraphPad Prism with the equation *v* = *k*_cat_[*S*]/(*K*_M_(1 + [*I*]/*K*_i_) + [*S*]) (where *v* = velocity, *k*_cat_ = catalytic rate constant, *K*_M_ = Michaelis-Menten constant, *K*_i_ = inhibitor constant) with the independent variables inhibitor concentration [*I*] and substrate concentration [*S*].

### In vitro SDH enzyme activity assay

SDH inhibition was tested with the MitoCheck Complex II Activity Assay Kit (Cayman). The kit provides reagents for measuring SDH activity in bovine heart mitochondria. Itaconate, citraconate and mesaconate were neutralized with NaOH (pH 7.4–7.7) and tested at concentrations of 10 mM, 3 mM, 1 mM, 0.25 mM, 50 µM and 10 µM. The SDH inhibitor disodium malonate was used as positive control. The assay was performed in a 96-well plate in triplicate according to the manufacturer’s instructions under inhibition of mitochondrial complexes I, III and IV by 1 µM rotenone, 10 µM antimycin A and 1 mM KCN, respectively.

### Cell lines and culture conditions

A human acute monocytic leukaemia cell line (THP1, DSMZ no. ACC 16) was cultured in RPMI 1640 (Gibco, catalogue no. 31870-025) supplemented with 10% heat-inactivated fetal bovine serum advanced (FBS-11A; Capricorn Scientific) and 1% GlutaMAX-I (100×; Gibco, catalogue no. 35050-038), at 5% CO_2_ and 37 °C. Some 5 × 10^5^ cells were seeded in 12-well plates (Falcon) and then differentiated into adherent macrophages. Cells were stimulated with 125 ng ml^−1^ phorbol 12-myristate 13-acetate (Sigma-Aldrich, catalogue no. P8139) for 48 h in RPMI complete medium before the medium was refreshed. Cells were then allowed to further differentiate for an additional day. Differentiation was verified morphologically via microscopy and by flow cytometry for expression of CD14 and CD11c. Human adenocarcinoma cells resembling type II alveolar epithelial cells (A549, ACC107, obtained from DSMZ) were propagated in DMEM medium supplemented with 10% FCS and 1% GlutaMAX-I. The human keratinocyte cell line HaCaT was provided by T. Werfel (Hannover Medical School)^31^ and cultured in RPMI 1640 (Gibco, catalogue no. 21875-034) supplemented with 10% FCS at 5% CO_2_ and 37 °C. *NRF2*^*–/–*^ HaCaT cells are described in ref. ^32^. All cell lines were tested for *Mycoplasma* contamination using the Venor GeM Classic Mycoplasma detection kit for conventional PCR (Minerva Biolabs, catalogue no. 11-1100).

### Uptake experiments

dTHP1 cells were incubated for 6 or 24 h with different concentrations of itaconate (0.125, 5, 10 and 25 mM), mesaconate or citraconate (5, 10, and 25 mM) in 1 ml of RPMI. After complete removal of medium and careful washing (one to three times) of cells and well borders with 1 ml of PBS, cells were extracted with 1 ml of ice-cold extraction buffer (acetonitrile/methanol/water, 2:2:1) containing internal standards, followed by mixing for 30 s and were kept at –20 °C for short-term storage and –80 °C for long-term storage. For the LPS/IFN-γ co-stimulation experiment, dTHP1 cells were treated with 200 ng ml^−1^ LPS (Sigma, catalogue no. L6511) and 400 U ml^−1^ human IFN-γ (PeproTech, catalogue no. 300-02) for 0, 6, 12, 18, 24, 30 and 48 h and then extracted with 1 ml of ice-cold extraction buffer as described previously^5^.

### Targeted inactivation of the *ACOD1* gene in THP1 cells by CRISPR–Cas9

We deleted a 2,962 bp fragment (7,347–10,308) spanning part of exon 4 and the whole of exon 5. THP1 cells were transiently electroporated with two EF1α-Cas9-2A-EGFP/U6-guideRNA expression plasmids (1250 V, 50 ms, 1 pulse; 5 × 10^6^ cells ml^−1^, 50 µg ml^−1^ each plasmid), using the Neon Transfection System (ThermoFisher Scientific)^33^, one containing a guide RNA directed against *ACOD1* exon 4 (5′-CCATGGATTTTGATGACACG-3′) and the other containing a guide RNA directed against the 3′ untranslated region of the *ACOD1* locus (5′-CATGAGCCTCAAGGTTTTAG-3′). Cells were sorted for enhanced green fluorescent protein expression after 18 h and seeded as single-cell colonies via limited dilution. After expansion, *ACOD1*^*–/–*^-deficient clones were identified by genomic PCR and Sanger sequencing. The knock-out was verified by confirming the absence of ACOD1 protein by immunoblot (Supplementary Fig. 6b) and the absence of itaconate synthesis by liquid chromatography with tandem mass spectrometry (LC–MS/MS) (Extended Data Fig. 2a).

### Anti-inflammatory and immunomodulatory effects of itaconate isomers

Some 5 × 10^5^ dTHP1 cells were infected with IVA (H1N1) strain PR8M at a multiplicity of infection of 1. In the case of treatments, the cells were pre-incubated for 12 h with pH-adjusted buffer containing itaconate, mesaconate, citraconate and/or 4-octyl itaconate at the concentrations indicated in the figures and were then incubated with the virus for 2 h in fresh medium to allow virus binding and entry into cells. The infection medium was subsequently replaced with fresh pH-adjusted medium containing the treating compound at the indicated concentration. Twelve hours after infection, cells were washed in buffer, pelleted and RNA was extracted for subsequent analysis of mRNA expression by quantitative PCR with reverse transcription (RT–qPCR), using the primer sequences listed in Supplementary Table 2. CXCL10 concentrations in cell supernatants were measured using the Human CXCL10 Standard ABTS ELISA Development Kit (PeproTech, catalogue no. 900-K39). Cytokine/chemokine concentrations in supernatants were measured using the human 27-plex cytokine panel (Bio-Rad, catalogue no. 171-A1112) as described in ref. ^10^, from which the following text was used: “Standard curves were generated with eight 2-fold serial dilutions starting from 32 ng/mL. 100 μL of assay buffer was added to each well of a microfiltration plate, followed by addition of 50 μL of beads suspension. After washing the beads with assay buffer, 50 μL of standard or the sample (supernatant) was added to each well and incubated for 30 min at room temperature (RT) with gentle shaking. 25 μL of antibody premix for detection was added into each well followed by incubation for 30 min at RT with gentle shaking. Three washing steps were performed using 50 μL of assay buffer in each well, followed by washing with streptavidin solution for 10 min at RT with shaking. Final washing with 125 μL of assay buffer was performed before quantification by using BIO-PLEX Manager software version 4.” IL-7 and IL-13 were excluded from further analyses due to concentrations below the limit of detection in most samples.

### Determination of viral titres

As described previously^34^, MDCK-II cells (American Tissue Culture Collection, catalogue no. CRL-2936) were cultured in 96-well plates and grown overnight at 37 °C in a humidified 5% CO_2_ incubator. The 90% confluent cells were washed once with PBS^++^ (1× PBS containing Ca^2+^, Mg^2+^_,_ 0.3% BSA (Sigma) and Penicillin-Streptomycin (Invitrogen)) and cells were infected with the 10-fold serial virus sample dilutions and incubated at room temperature for 1 h. Virus inoculum was aspirated and 150 µl of 1.25% Avicel containing 1 µg of N-p-tosyl-L-phenylalanyl chloromethyl ketone-treated trypsin per ml was added to each well and incubated at 37 °C, 5% CO_2_ for 24 h. Cells were then fixed at room temperature for 1 h with PBS^++^ containing 3.7% formaldehyde and 1% Triton X-100. Cells were washed for three times with PBS/Tween-20 (0.05%), then incubated with primary antibody (anti-IAV-NP mouse monoclonal IgG, hybridoma supernatant diluted 1:100; provided by S. Ludwig, University of Münster) at room temperature for 1 h. Cells were then washed three times and incubated with goat anti-mouse horseradish peroxidase (HRP)-conjugated IgG (Invitrogen A16072) at room temperature for 1 h. 3-Amino-9-ethylcarbazole (Sigma) was used as the substrate for immunostaining and incubated at room temperature for 30–60 min. After clear development of the red-stained foci, they were counted to determine the viral titre in focus-forming units (FFU) using the equation FFU ml^−1^ (stock) = (1/virus dilution) × (number of foci) × (dilution factor).

### Human lung tissue explant model

Use of human tissues was approved by the Ethics Committee of Hannover Medical School (file no. 2923-2015) and all donors gave informed consent for use of the tissue for research purposes. Explanted lung tissue from patients with a clinical indication for lung transplantation was divided into pieces of approximately 30 mg and cultured and infected essentially as described in ref. ^10^. Tissues were kept in RPMI buffer for up to 20 h before the start of the experiment. Tissue pieces were pretreated for 14–19 h with pH-adjusted buffer containing compound or buffer only and then incubated in RPMI infection medium containing IAV (2 × 10^5^ FFU ml^−1^) and compound or buffer only for another 24 h. Three pieces each from five donors (three emphysema, three idiopathic pulmonary fibrosis; two male, three female; age 58–66 years) were used.

### Immunoblotting

Cells were washed once in ice-cold PBS and lysed in ice-cold RIPA buffer (containing protease and phosphatase inhibitor). Upon quantification of protein by Bradford assay an equal volume of 2× Laemmli sample buffer was added. Samples were heat-denatured at 95 °C for 10 min. Protein extracts were resolved by gel electrophoresis and transferred to a 0.2 µm NC nitrocellulose blotting membrane (GE Healthcare, catalogue no. 10600004). Non-specific binding was blocked with 5% non-fat dry milk, and bands were visualized by enhanced chemiluminescence with primary antibodies specific to STAT1 (Santa Cruz Biotechnology, catalogue no. sc-345; diluted 1:500), phospho-STAT1 (Cell Signaling, catalogue no. 9167S; diluted 1:1,000), NRF2 (Cell Signalling, catalogue no. 12721S; diluted 1:1,000), followed by incubation with goat anti-rabbit IgG–HRP (Southern Biotech, catalogue no. 4030-05). β-Actin was visualized using HRP-conjugated anti-β-actin antibody (Abcam, catalogue no. ab49900) or β-actin antibody (C4) mouse monoclonal antibody IgG1 (Santa Cruz Biotechnology, catalogue no. sc-47778). Membranes were developed using Amersham ECL Prime Western Blotting Detection Reagent (GE Healthcare, catalogue no. RPN2232). An iNTAS western blot imager (iNTAS Science Imaging) was used for imaging of the membrane.

### ROS measurements

dTHP1 cells were seeded at a density of 5 × 10^5^ cells per well in a 12-well plate. Cells were pre-incubated with the treatments for 12 h, infected with IAV (multiplicity of infection of 1) and incubated with fresh medium containing the treatments. Mitochondrial ROS levels were measured 12 h after infection. Cells were incubated for 5 min with medium containing 5 μM of MitoSox Red (mitochondrial superoxide indicator; ThermoFisher Scientific, catalogue no. M36008) and then washed with PBS. Cells were then resuspended in cold PBS and mitochondrial ROS levels were measured via the phycoerythrin channel using a BD LSR-II flow cytometer.

### Assessment of NRF2 signalling

HaCaT cells (1.5 × 10^5^) were seeded in 12-well plates overnight, then treated with the indicated concentrations of itaconate, mesaconate and citraconate for 16 or 17.5 h. Cells were then washed in buffer, pelleted and RNA was extracted for mRNA measurements by RT–qPCR using the primer sequences listed in Supplementary Table 2. NRF2 stabilization was assessed by immunoblot (see above).

### MTT assay

Some 2.5 × 10^4^ THP1 cells were seeded in a 96-well plate (Falcon) and then differentiated into adherent macrophages. The cells should be 80%–100% confluent before starting the assay. MTT reagent stock (Life Technologies, catalogue no. M6494; 5 mg ml^−1^ in PBS) was diluted 1:10 in 37 °C RPMI complex medium. After removal of supernatant, 50 µl of the prepared dilution was added to the cells and incubated at 37 °C for 20–60 min while periodically observing the colour in the cells. The reagent was removed when staining was completed, and 50 µl of dimethylsulfoxide (Merck) was added. After mixing on a shaker for 5–15 min (until the solution was homogeneous), the colour change was quantified by enzyme-linked immunosorbent assay reader (BioTek Synergy 2), using 540/570 nm as the measurement wavelength and 630 nm as the reference wavelength.

The mouse model was performed essentially as described in ref. ^35^, from which parts of the following text were used: “All animal procedures were approved by the University of Luxembourg Animal Experimentation Ethics Committee and by appropriate government agencies. The animal work of the present study has been conducted and reported in accordance with the ARRIVE (Animal Research: Reporting of in vivo Experiments) guidelines to improve the design, analysis and reporting of research using animals, maximizing information published and minimizing unnecessary studies. Three-to-four-month-old C57BL/6N male and female mice were obtained from Charles River Laboratories (France). Mice were housed in 12 h light/dark cycle, with sterile food and water ad libitum. The mice were specific pathogen free, housed in individually ventilated cages at a maximum of 5 per cage and maintained at a temperature of 22 °C and a relative humidity of 55%. They were kept on autoclaved corn cob bedding and fed with SAFE Diets irradiated at a min of 25 kGy. The watering system consisted of reverse osmosis water with 2 ppm of chlorine. Mice were treated with a single intraperitoneal injection of LPS (4 μg LPS/g body weight) or PBS as vehicle control. Mice were deeply anaesthetized with a combination of ketamine (100 mg/mL; Nimatek Vet) and dorbene (medetomidine hydrochloride; 1 mg/mL; Dorbene Vet) 0, 12, 24 and 48 h after LPS injection. Spleens were dissected following transcardiac perfusion with ice-cold PBS, collected in ice-cold HBSS (Gibco/Life Technologies) with 1 M HEPES (Gibco/Life Technologies) and 0.5% D-(+)-glucose (Sigma-Aldrich) and then stored in liquid nitrogen.”

### Quantification of itaconate isomers, TCA intermediates and lactate

Measurements were performed according to our validated high-performance liquid chromatography-tandem mass spectrometry (HPLC–MS/MS) assay^5^. Briefly, samples were extracted in 1,000 µl of extraction reagent (methanol/acetonitrile/water with a final ratio of 2:2:1 v/v/v; spiked with 0.1 µM ^13^C_2_-citrate and ^13^C_5_-itaconate, 0.2 µM ^13^C_6_-*cis*-aconitate and ^13^C_4_-succinate, and 1 µM ^13^C_3_-lactate as internal standards). The suspensions were transferred to 2 ml safe-lock reaction tubes (Eppendorf, catalogue no. 0030120094), vortexed for 30 s and frozen at −20 °C overnight to complete protein precipitation. Subsequent sample preparation and HPLC–MS/MS assay using a Kinetex C18 reversed-phase column (Phenomenex, catalogue no. 00D 4462 Y0) on a Nexera chromatography system (Shimadzu) coupled to a QTRAP5500 triple quadrupole/linear ion trap mass spectrometer (Sciex) were essentially performed as described^5^.

### Targeted metabolite profiling

Cell metabolites were extracted as described in ref. ^36^, using 6 × 10^6^ cells per sample. The concentrations of amino acids (*n* = 20), amino acid metabolites (*n* = 30), biogenic amines (*n* = 9), succinate and lactate were measured on an AB SCIEX 5500 QTrap mass spectrometer (Ab Sciex), using the MxP Quant 500 kit (Biocrates Life Sciences) according to the manufacturer’s protocols (https://biocrates.com/mxp-quant-500-kit). Ratios and sums of analytes (‘metabolite indicators’) were calculated using the MetaboIndicator software (Biocrates).

### Mito Stress Test by Seahorse

Oxygen consumption rate was determined using a Seahorse XF-96 Analyzer (Agilent) and the Mito Stress Test kit (Agilent, catalogue no. 103015-100) following the manufacturer’s protocols. Briefly, 2.5 × 10^4^ THP1 cells were plated on Agilent Seahorse XF96 cell culture microplates (part no. 101085-004) and differentiated with phorbol 12-myristate 13-acetate as described above. On the day of the assay, the cell culture medium was changed to Seahorse XF RPMI medium (Agilent, catalogue no. 103576-100) containing 10 mM glucose (Agilent, catalogue no. 103577-100), 1 mM pyruvate (Agilent, catalogue no. 103578-100) and 2 mM glutamine (Gibco, catalogue no. 35050-038), and placed in a 37 °C non-CO_2_ incubator for 45–60 min before the assay. Upon loading to the Seahorse XF-96 Analyzer, sequential in situ incubation of components was performed as follows: baseline measurement for 18 min, 1.5 µM oligomycin (Agilent) for 18 min, 1 µM carbonyl cyanide-*p*-trifluoromethoxyphenylhydrazone (Agilent) for 18 min and 0.5 µM rotenone/antimycin A (Agilent) for 18 min. Oxygen consumption rate data were analysed with Wave v.2.2.1 software (Agilent).

### Electrophilicity assessment via reaction with glutathione

Itaconic, mesaconic or citraconic acid (13.0 mg, 100 µM) or oxaloacetic acid (13.2 mg, 100 µM) were added in parallel to a set of four 2-ml Eppendorf tubes containing reduced l-glutathione (30.7 mg, 100 µM). The reaction mixtures were dissolved in Milli-Q water (1 ml) and were stirred at room temperature for 2 h. Aliquots of 10 µl were transferred to LC–MS vials containing Milli-Q water (490 µl), acetonitrile (495 µl) and diphenhydramine hydrochloride (5 µl of 10 µM solution in acetonitrile) as the internal standard. The vials were capped, vortexed and subjected to ultra-performance liquid chromatography–high-resolution mass spectrometry analysis using a Dionex UltiMate 3000 UHPLC^+^ focused/Thermo Scientific Q Exactive Focus Orbitrap LC–MS/MS system (ThermoFisher Scientific). This system consists of Dionex UltiMate 3000 RS pump, RS autosampler, RS column compartment, diode array detector and quadrupole–Orbitrap mass spectrometer, as well as the standard software Xcalibur v.4.4.16.14 for operation. A RP EC 150/2 NUCLEODUR C18 Pyramid, 3 µm (150 mm × 2 mm) column (Macherey-Nagel) was used as the stationary phase, and a binary solvent system A and B (A = water with 0.1% formic acid; B = acetonitrile with 0.1% formic acid) was used as the mobile phase. In a gradient run, the percentage of B was kept constant at an initial concentration of 1% from 0 to 2 min, then increased from 1% at 2 min to 60% at 8.5 min, then to 95% at 9.5 min and kept at 95% for 0.4 min. The injection volume was 2 μl, and the flow rate was set to 250 μl min^−1^. The column temperature was 40 °C, and ultraviolet tracing was acquired at wavelength of 254 nm. High-resolution mass spectrometry data were recorded on a Thermo Scientific Q Exactive Focus Orbitrap system. Mass spectra were acquired in positive mode from 100 to 1000 *m*/*z*. MS analysis by heated electrospray ionization was carried out at a spray voltage of 3800 V, and an ion transfer tube temperature of 350 °C.

### Computational chemistry

All computational work was performed using Molecular Operating Environment (MOE), v.2020.09, Chemical Computing Group ULC, 910–1010 Sherbrooke St. W. Montreal, Quebec, H3A 2R7, Canada. The computational procedure was adapted from a reported protocol^37^ with slight modifications.

### Preparation of ligands and protein structures

The two-dimensional structures of itaconic acid, mesaconic acid and citraconic acid were sketched using ChemDraw professional 19.0 and were imported into the MOE window. The compounds were subjected to an energy minimization up to a gradient of 0.001 kcal mol^−1^ Å^2^ using the MMFF94x force field and R-field solvation model, then saved as an mdb file. The predominant protonation status of the compounds in aqueous medium at pH 7 was calculated via the compute | molecule | wash command in the database viewer window. X-Ray crystal structures of human SDHA in complex with the cofactor oxaloacetate (PDB ID: 6VAX)^38^, porcine mitochondrial respiratory complex II, containing oxaloacetate (PDB ID: 3SFD)^39^, human ACOD1 (PDB ID: 6R6U)^2^ and mouse ACOD1 (PDB ID: 6R6T)^2^ were used for the molecular docking studies. Potential was set up to Amber10:EHT as a force field and R-field for solvation. Addition of hydrogen atoms, removal of water molecules more than 4.5 Å from the ligand or receptor, correction of library errors and tethered energy minimization of the binding site were performed via the QuickPrep module.

### Structural modelling

The binding site was set to dummy atoms which were identified by the site finder command. The amino acid residues delineating the binding site of oxaloacetate/succinate in the active site of the SDHA subunit were selected. For ACOD1, the binding site was defined according to the putative active site^2^. Docking placement was triangle matcher with an induced fit refinement option. The first scoring function was alpha HB with 1,000 poses, followed by a refinement score London dG with 10 poses.

### Calculation of electrophilicity descriptors

In the database viewer window, molecular descriptors were calculated for all entries via activating the compute panel and choosing descriptors calculate option. The energies (eV) of the lowest unoccupied molecular orbital and the highest occupied molecular orbital were calculated using the semi-empirical Austin Model 1 (AM1) Hamiltonian by the quantum chemistry program MOPAC v.7.0 included in MOE.

### Similarity study of dicarboxylates

In the database containing the dicarboxylates, the two-dimensional fingerprint BIT_MACCS (166 public MDL MACCS structural keys, bit packed) was calculated for all entries. Succinate was selected as a query structure and was sent to the MOE window. In the database viewer window, the similarity search was performed by choosing compute | fingerprint | search command. The fingerprint system was set to BIT_MACCS and the Tanimoto coefficient (*T*_C_) as a similarity metric. *T*_C_ values range from 0 (no similarity) to 1 (complete similarity). The similarity search for *cis*-aconitate was performed in the same manner using the piDAPH3 (3-point pharmacophore based on the 3D conformation, considering the pi system, donor and acceptor atomic properties) as fingerprint and *cis*-aconitate as a query structure.

### Statistics and bioinformatics

Significance of differences between more than two groups was assessed with one-way analysis of variance (ANOVA) followed by Dunnett’s multiple comparisons test. Unpaired *t*-test was used to assess significance of differences between two groups of *n* *=* 4 or *n* *=* 3, the Mann–Whitney *U* test for comparison of medians of non-normally distributed data when *n* ≥ 7. We adjusted for testing of multiple hypotheses as indicated in the figure legends. Significance was defined as a *P* value or false discovery rate ≤0.05 using the following symbols: *≤0.05, **≤0.01, ***≤0.001 and ****≤0.0001. In the cellular experiments, *n* always refers to biological replicates, for example cells of the same line in that were cultivated in a separate well but subjected to the same experimental manipulations as the other replicates. Unless stated otherwise, data are shown as means, with error bars indicating s.d. and statistical analyses were performed using GraphPad Prism v.9.3.1 (GraphPad Software). For PCA, values were log_2_-transformed and then analysed with MetaboAnalyst v.5.0 (https://www.metaboanalyst.ca). Venn diagrams were drawn using jvenn (http://jvenn.toulouse.inra.fr/app/example.html).

### Reporting summary

Further information on research design is available in the Nature Research Reporting Summary linked to this article.

### Supplementary information

Supplementary Information Supplementary Figs. 1–6 and Tables 1 and 2.

Reporting Summary

Supplementary Data 1 Source data of LC−MS/MS amino acid analysis pertaining to Extended Data Fig. 5 and Supplementary Figs. 3 and 4.

Supplementary Data 2 Source data of microbead multiplex cytokine/chemokine analysis pertaining to Fig. 2 and Extended Data Fig. 6.

Supplementary Data Source data blots for Supplementary Fig. 6b.

### Source data

Source Data Fig. 2 Uncropped membrane images of immunoblots, Fig. 2a, j and k.

## Figures and Tables

**Extended Data Table 1:**
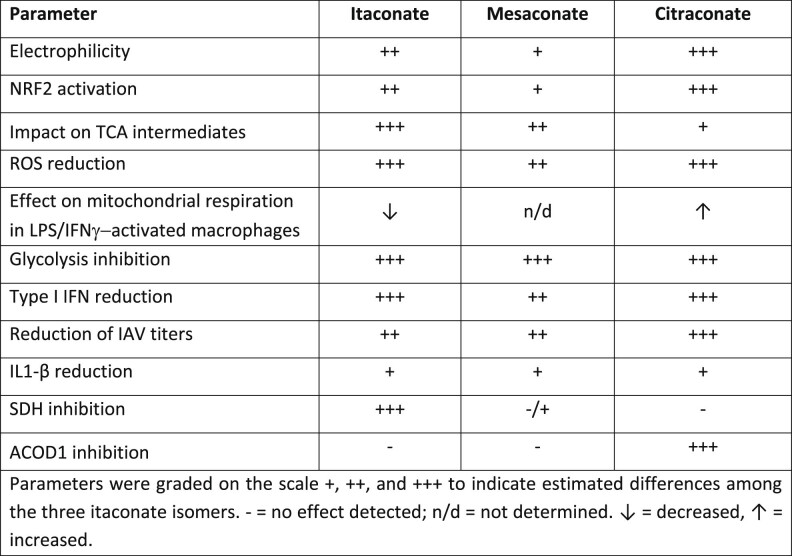
Comparison of biochemical properties and effects of the itaconate isomers

## Data Availability

Source data are provided with this paper. The source data underlying the amino acid-related analysis shown in Extended Data Fig. 5 and Supplemental Figs. 3 and 4 are available in Supplementary Data 1. The raw data underlying the multiplex cytokine/chemokine analysis shown in Fig. 2 and Extended Data Fig. 6 are available in Supplementary Data 2. The data that support the other plots within this paper and other findings of this study are available from the corresponding author upon reasonable request.
