# Supplementary material for: Citraconate inhibits ACOD1 (IRG1) catalysis, reduces interferon responses and oxidative stress, and modulates inflammation and cell metabolism
Source: Nat Metab. 2022 Jun 2;4(5):534–46. doi: 10.1038/s42255-022-00577-x (PMC9170585; doi:10.1038/s42255-022-00577-x)
Supplement: Supplementary file 1 — Supplementary Figs. 1–6 and Tables 1 and 2. [file 42255_2022_577_MOESM1_ESM.pdf]

nature portfolio

https://doi.org/10.1038/s42255-022-00577-x

Supplementary information

Citraconate inhibits ACOD1 (IRG1) catalysis, reduces interferon responses and oxidative stress, and modulates inflammation and cell metabolism

In the format provided by the
authors and unedited

Supplementary Information

Citraconate inhibits ACOD1 (IRG1) catalysis, reduces interferon responses and oxidative stress, and modulates inflammation and cell metabolism

Chen F, Elgaher WAM, Winterhoff M, Büssow K, Waqas F, Graner E, Pires-Afonso Y, Casares Perez L, De la Vega L, Sahini N, Czichon L, Zobl W, Zillinger T, Shehata M, Pleschka S, Bähre H, Falk C, Michelucci A, Schuchardt S, Blankenfeldt W, Hirsch AKH, Pessler F

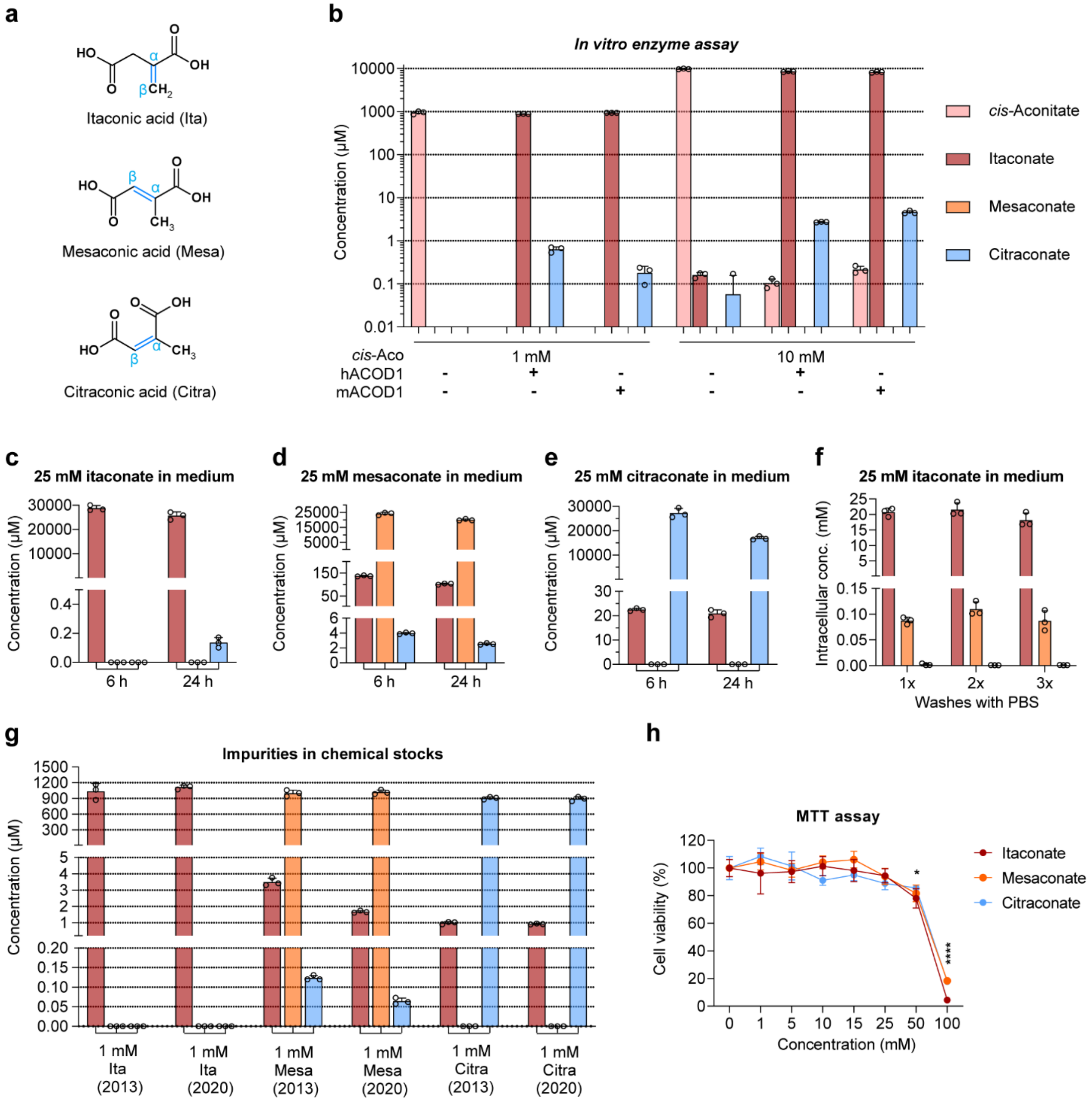

Figure S1. Lack of interconversion among the three itaconate isomers in cell-free conditions. a, Structures of the isomers itaconic, mesaconic, and citraconic acid. α and β indicate positions of unsaturation, where the β position is the most electrophilic site for Michael reaction. **b, ACOD1 catalysis does not produce mesaconate and only negligible amounts of citraconate.** Recombinant human or murine ACOD1 was incubated with 1 or 10 mM *cis*-aconitate in a cell-free assay, and itaconate, mesaconate, and citraconate accumulation was measured by HPLC-MS/MS. The expected high amounts of itaconate were detected, but no mesaconate. There were very

small amounts of citraconate (<0.07% of itaconate) indicating that it is a very minor by-product of ACOD1 activity in vitro. *n*=3 independent assays, means ± s.d. **c–e, Absence of catabolism of the three itaconate isomers in cell culture medium.** Increasing concentrations of itaconate, mesaconate or citraconate were incubated in RPMI complex medium at 37°C for 6 h or 24 h, and concentrations of all three isomers were measured by HPLC-MS/MS. Concentrations did not decrease, but low-level contamination of citraconate and mesaconate by itaconate was observed. *n*=3 independent incubations, mean ± s.d. **f, Effect of washing cells on detection of intracellular itaconate isomer concentrations.** dTHP1 cells were incubated in the presence of 25 mM itaconate for 6 h, the medium was aspirated, and cells were washed 1–3 times in ice-cold PBS. Cellular itaconate and mesaconate concentrations did not change after any number of washes, demonstrating that the measured compound was intracellular and not adherent to the cell surface. Citraconate was not detected. *n*=3 biological replicates, mean ± s.d. **g, Impurities in stocks of itaconic, mesaconic, and citraconic acid prepared from pure commercially available compounds.** Two different batches of each of the isomers were analyzed by HPLC-MS/MS for concentrations of the respective two other isomers. Small fractions of itaconate were detected in mesaconate and citraconate stocks, and very low concentrations of citraconate in mesaconate stocks that actually were near the LLOQ of the assay (0.049 µM). *n*=3 aliquots of each batch, mean ± s.d. **h, Determination of nontoxic concentrations of the three isomers.** dTHP1 cells were incubated with increasing concentrations of each isomer and cell viability was assessed by MTT assay. 25 mM was identified as the highest nontoxic concentration of all isomers. *n*=3 biological replicates, mean ± s.d. One-way ANOVA followed by Dunnett's multiple comparisons test. * $P \leq 0.05$, ** $P \leq 0.01$, *** $P \leq 0.001$, **** $P \leq 0.0001$.

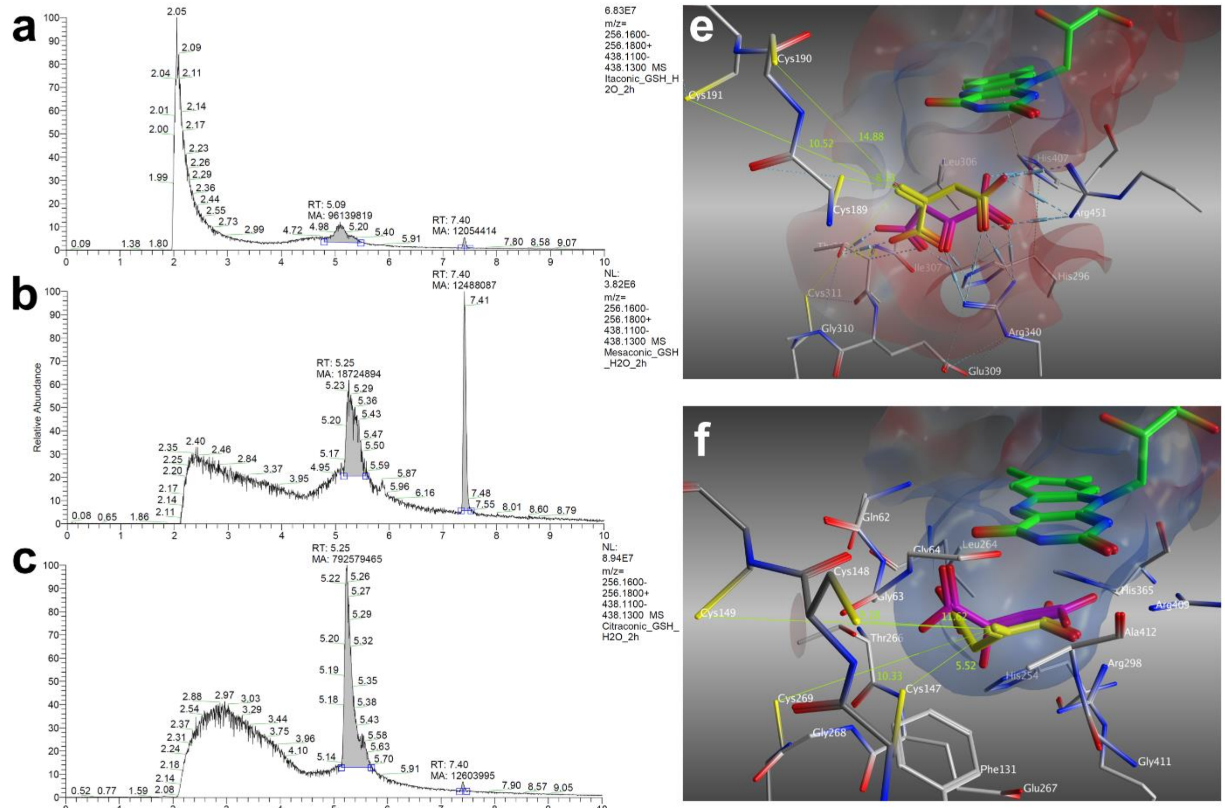

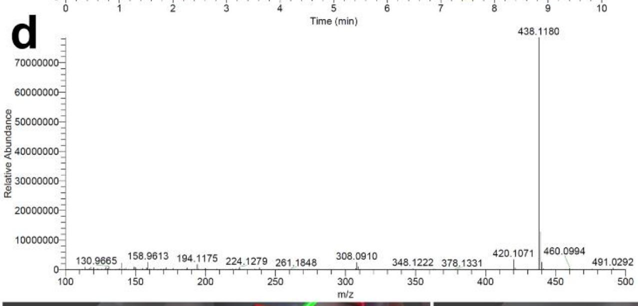

g

Compound	Tanimoto coefficient (T_C) (FP:BIT_MACCS)	Binding energy score (S) (kcal/mol)
**Itaconate**	**0.68**	**–406 to –380**
**Mesaconate**	**0.56**	**–365 to –340**
**Citraconate**	**0.56**	**–381 to –360**
**Oxaloacetate**	**0.73**	**–403 to –383**

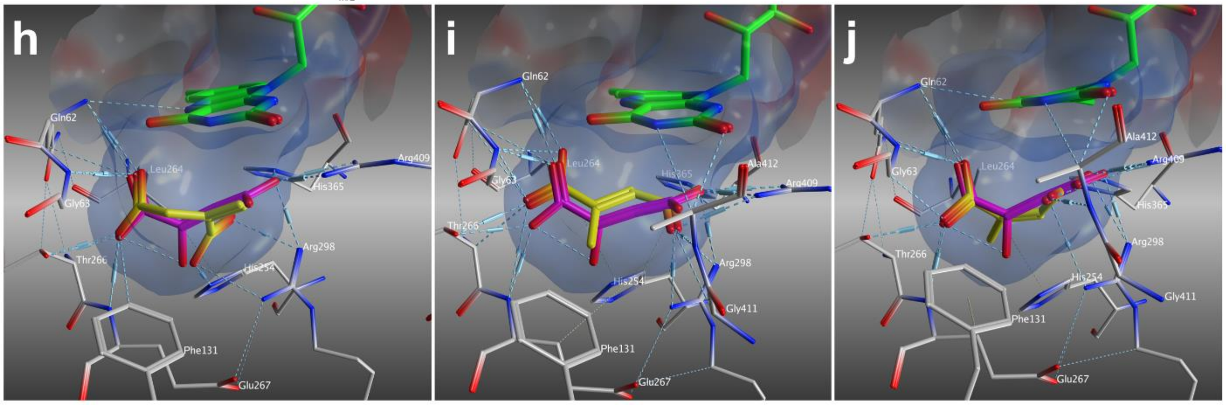

Figure S2. Extracted ion chromatogram of itaconate (**a**), mesaconate (**b**), and citraconate (**c**) incubated with GSH showing the formation of Michael adduct at t_R 5.09 and 5.25 min, respectively, compared to diphenhydramine as an internal standard at t_R 7.40 min. **d,** ESI HRMS showing the Michael addition product $[M+H]^+$ = 438.1182 Da from the citraconate and GSH

reaction. **e** and **f**, Potential binding poses of itaconate (yellow) compared to oxaloacetate (magenta) in the succinate binding site of human SDHA (PDB ID: 6VAX)[1], and porcine SDHA (PDB ID: 3SFD)[2], respectively. The nearest cysteine residues (Cys189, 190, 191, and 311) for human SDHA (**e**) and (Cys147, 148, 149 and 269) for porcine SDHA (**f**) are located at distances of 5.5 to 14.9 Å from the electrophilic β-carbon of the SDHA-bound itaconate, which are longer than required for a Michael reaction to occur (4.0–4.5 Å). Moreover, the thiol groups are less accessible and are placed at disfavored angles to the olefinic bond of itaconate (70–73° and 135–175° vs an optimum of 94–115°). Electrostatic protein surface at the active site: positive (blue), negative (red), neutral (white). Flavin adenine dinucleotide (FAD, green). **g, Similarity to succinate and binding energies of the dicarboxylates to SDHA.** Potential binding modes of itaconate (**h**), mesaconate (**i**), and citraconate (**j**) (all yellow) compared to oxaloacetate (magenta) in the succinate binding site of porcine SDHA (PDB ID: 3SFD). Binding of itaconate was attained entirely through a network of electrostatic attractions, i.e., hydrogen bonds and salt bridges, (dashed lines) between the C1- and C4-carboxyl groups and the active site residues (Gln62, Gly63, His254, Thr266, Glu267, Arg298, and His365). Similar to itaconate, the C1- and C4-carboxyl groups of mesaconate and citraconate are the exclusive moieties responsible for binding through hydrogen bonds and ionic interactions (dashed lines). However, both molecules adopt mainly rigid and planar configurations, which may hamper an optimum interaction.

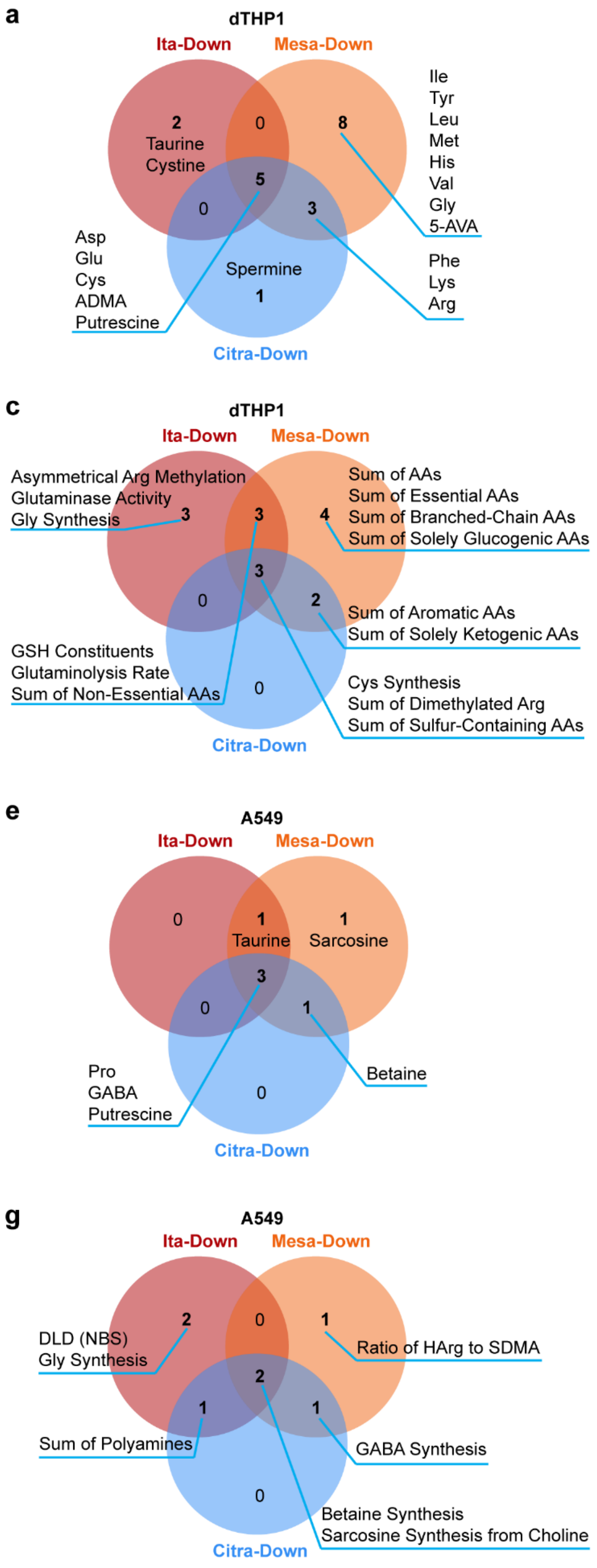
a
dTHP1
Ita-Down
Mesa-Down
2
Taurine
Cystine
0
8
Ile
Tyr
Leu
Met
His
Val
Gly
5-AVA
5
0
3
Asp
Glu
Cys
ADMA
Putrescine
Spermine
1
Phe
Lys
Arg
Citra-Down
b
dTHP1
Ita-Up
Mesa-Up
0
0
0
1
2
0
Asn
Pro
GABA
Betaine
2
t4-OH-Pro
Citra-Up
c
dTHP1
Ita-Down
Mesa-Down
Asymmetrical Arg Methylation
Glutaminase Activity
Gly Synthesis
3
3
4
Sum of AAs
Sum of Essential AAs
Sum of Branched-Chain AAs
Sum of Solely Glucogenic AAs
3
0
2
Sum of Aromatic AAs
Sum of Solely Ketogenic AAs
GSH Constituents
Glutaminolysis Rate
Sum of Non-Essential AAs
0
Cys Synthesis
Sum of Dimethylated Arg
Sum of Sulfur-Containing AAs
Citra-Down
d
dTHP1
Ita-Up
Mesa-Up
0
2
1
b-Ala Synthesis
IDO Activity
Spermidine Synthesis
4
Taurine Synthesis
1
0
DLD (NBS)
Asn Synthesis
GABA Synthesis
Pro Hydroxylation
3
HCys Synthesis
Betaine Synthesis
Carnosine Synthesis
Citra-Up
e
A549
Ita-Down
Mesa-Down
0
1
Taurine
1
Sarcosine
3
0
1
Pro
GABA
Putrescine
0
Betaine
Citra-Down
f
A549
Ita-Up
Mesa-Up
Ala
Asp
Ser
Thr
ADMA
5
0
0
1
0
0
0
Kynurenine
Citra-Up
g
A549
Ita-Down
Mesa-Down
2
0
1
DLD (NBS)
Gly Synthesis
Ratio of HArg to SDMA
2
1
1
Sum of Polyamines
GABA Synthesis
0
Betaine Synthesis
Sarcosine Synthesis from Choline
Citra-Down
h
A549
Ita-Up
Mesa-Up
1
1
1
Pro Hydroxylation
Spermine Synthesis
Spermidine Synthesis
0
0
1
IDO Activity
0
Citra-Up

64

Figure S3. Shared and unique effects of the itaconate isomers on amino acid metabolism in IAV infected dTHP1 and A549 cells. Analysis of 59 amino acid-related analytes and their 66 metabolite indicators, same data set as in **Extended Data Fig 5.** Metabolite indicators are sums or ratios computed by MetaboIndicator™ software (Biocrates) that reflect mean abundance of groups of metabolites or predict activity of selected enzymes or pathways. **a–h, Venn diagrams showing analytes and metabolite indicators that are differentially abundant** (unpaired t-test, FC >1.3, FDR ≤0.05) in IAV-infected dTHP1 (**a–d**) and A549 (**e–h**) cells due to treatment with itaconate, mesaconate or citraconate. The intersects identify analytes or indicators that are commonly altered by one, two or all three isomers. Downregulated analytes and indicators for dTHP1 (**a,c**) and A549 (**e,g**) cells; Upregulated analytes and indicator for dTHP1 (**b,d**) and A549 (**f,h**) cells.

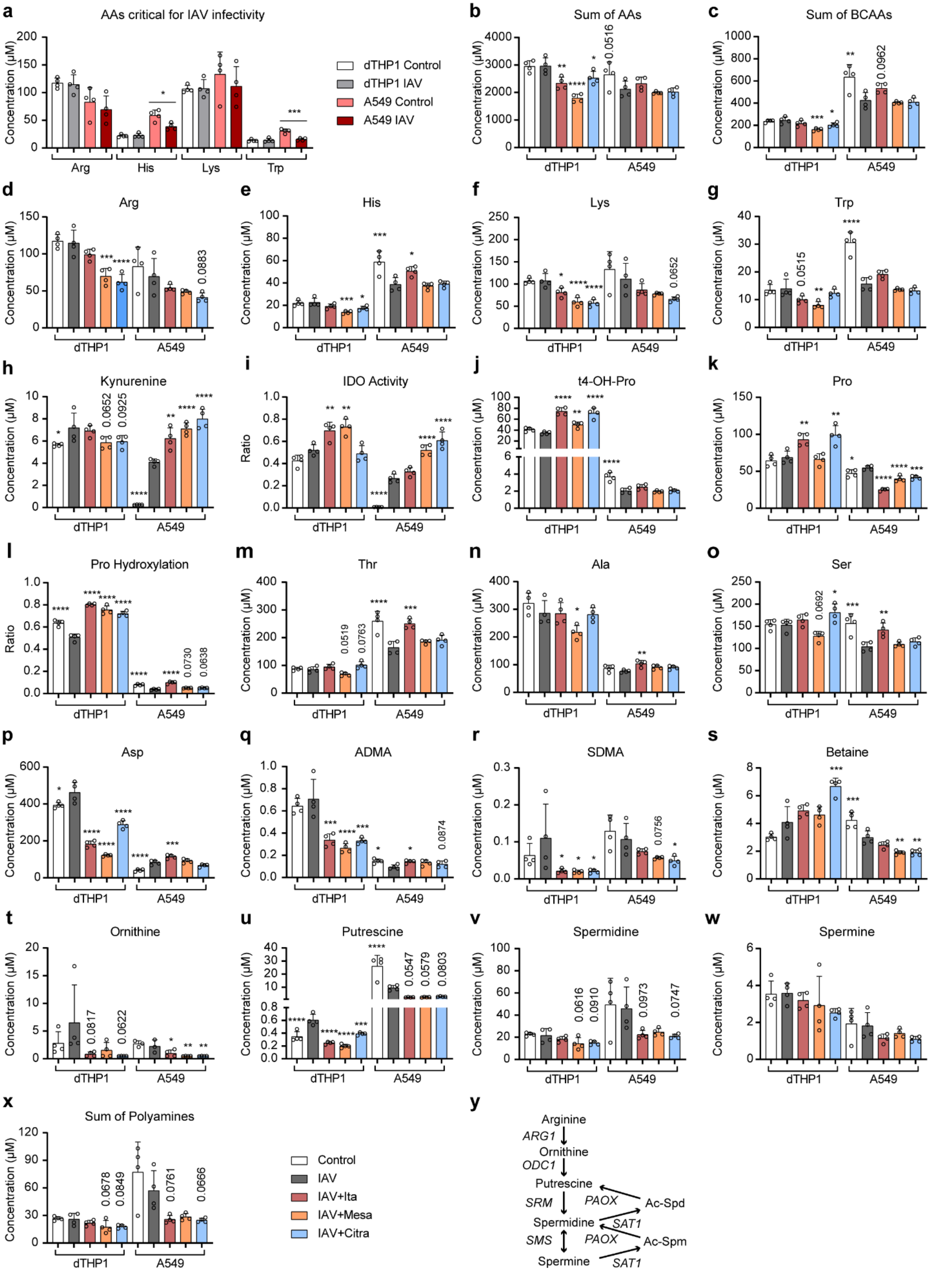
a
AAs critical for IAV infectivity
Concentration (μM)
Arg
His
Lys
Trp
dTHP1 Control
dTHP1 IAV
A549 Control
A549 IAV
b
Sum of AAs
c
Sum of BCAAs
d
Arg
e
His
f
Lys
g
Trp
h
Kynurenine
i
IDO Activity
Ratio
j
t4-OH-Pro
k
Pro
l
Pro Hydroxylation
m
Thr
n
Ala
o
Ser
p
Asp
q
ADMA
r
SDMA
s
Betaine
t
Ornithine
u
Putrescine
v
Spermidine
w
Spermine
x
Sum of Polyamines
dTHP1
A549
Control
IAV
IAV+Ita
IAV+Mesa
IAV+Citra
y
Arginine
ARG1
Ornithine
ODC1
Putrescine
SRM
PAOX
Ac-Spd
Spermidine
SAT1
SMS
PAOX
Ac-Spm
Spermine
SAT1

Figure S4 a-x, **Concentrations of the indicated amino acid-related analytes and calculated values of the metabolite indicators. y**, **Schematic of the polyamine pathway.** Same data set as in **Extended Data Fig. 5** and **Fig. S3**. Abbreviations and definitions: 5-AVA, 5-Aminovaleric acid; alpha-AAA, α-Aminoadipic acid; ADMA, asymmetric dimethyl arginine; 1-Met-His, 1-Methylhistidine; 3-Met-His, 3-Methylhistidine; ARG1, arginase 1; BCAAs, branched chain amino acids; DLD, dihydrolipoamide dehydrogenase; GABA, γ-aminobutyric acid; GSH = tripeptide glutathione; HCys, homocysteine; IDO (indoleamine 2,3,-dioxygenase) activity = kynurenine/Trp; proline hydroxylation = t4-OH-Pro/Pro; ODC1, ornithine decarboxylase; SRM, spermidine synthase; SMS, spermine synthase; PAOX, polyamine oxidase; SAT1, spermidine/spermine acetyltransferase; SDMA = symmetric dimethyl arginine; Sum of AA = sum of the measured 20 amino acids; sum of BCAAs = sum of branched chain amino acids (Leu+Ile+Val); sum of polyamines = sum of putrescine+spermidine+spermine; t4-OH-Pro, trans-4-hydroxyproline. $n=4$ biological replicates, mean ± s.d. One-way ANOVA followed by Dunnett's multiple comparisons test. * $P \leq 0.05$, ** $P \leq 0.01$, *** $P \leq 0.001$, **** $P \leq 0.0001$.

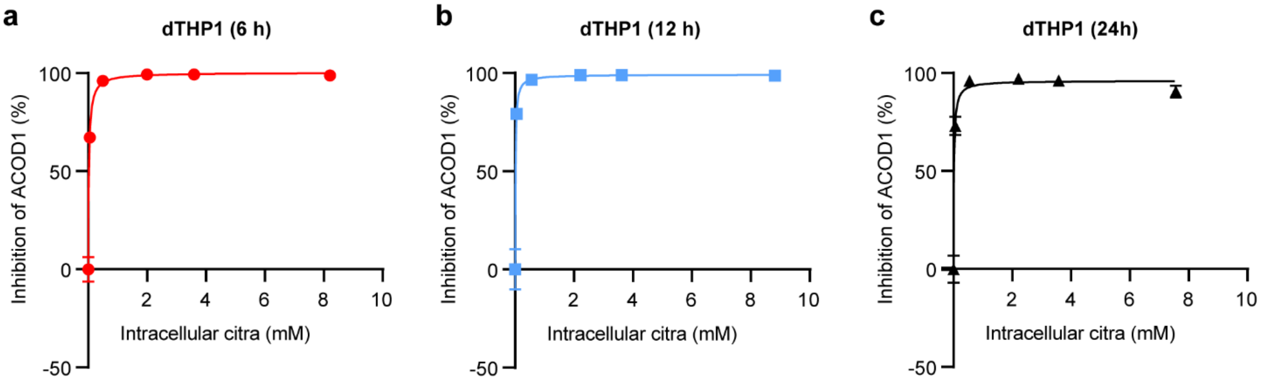

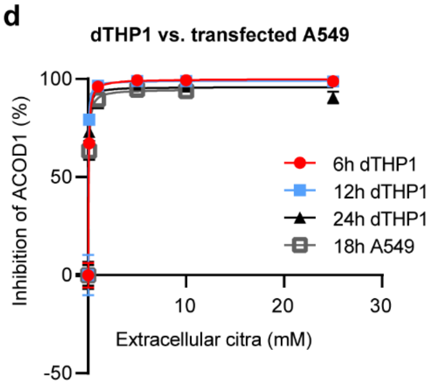

e

	IC_{50} for ACOD1 inhibition	
	**Intracellular citra (µM)**	**Extracellular citra (µM)**
**dTHP1 (6h)**	27.22 (95 % CI: 22.83 - 32.16)	48.46 (95 % CI: 40.75 - 57.11)
**dTHP1 (12h)**	14.19 (95 % CI: 9.697 - 19.33)	25.27 (95 % CI: 17.28 - 34.39)
**dTHP1 (24h)**	19.05 (95 % CI: 12.77 - 26.46)	30.35 (95 % CI: 20.50 - 41.95)
**A549 (18h)**		49.62 (95 % CI: 38.17 - 63.20)

Figure S5. Determination of IC_{50} values (ACOD1 enzyme activity) for extracellular and intracellular citraconate. dTHP1 cells were stimulated with LPS (200 ng/mL) and IFNγ (400 U/mL) for the indicated durations in the absence of presence of 0.1, 1, 5, 10, and 25 mM citraconate. Unstimulated dTHP1 cells treated for 6 h were used as additional control. Intracellular citraconate and itaconate concentrations were measured by LC-MS/MS. IC_{50} values were calculated using the Nonlin fit function in GraphPad Prism 9.3.1. **a-c**, ACOD1 inhibition in dependence of intracellular citraconate concentrations at 6, 12 and 24 h after LPS/IFN-γ stimulation. **d**, ACOD1 inhibition in dependence of extracellular citraconate at 6, 12 and 24 h after LPS/IFN-γ stimulation compared with IC_{50} of extracellular citraconate for hACOD1 (18 h) in the transfection experiment shown in **Fig. 3d**. *n*=3 biological replicates. **e**, Summary of the IC_{50} values derived from the curves shown in **a-d**.

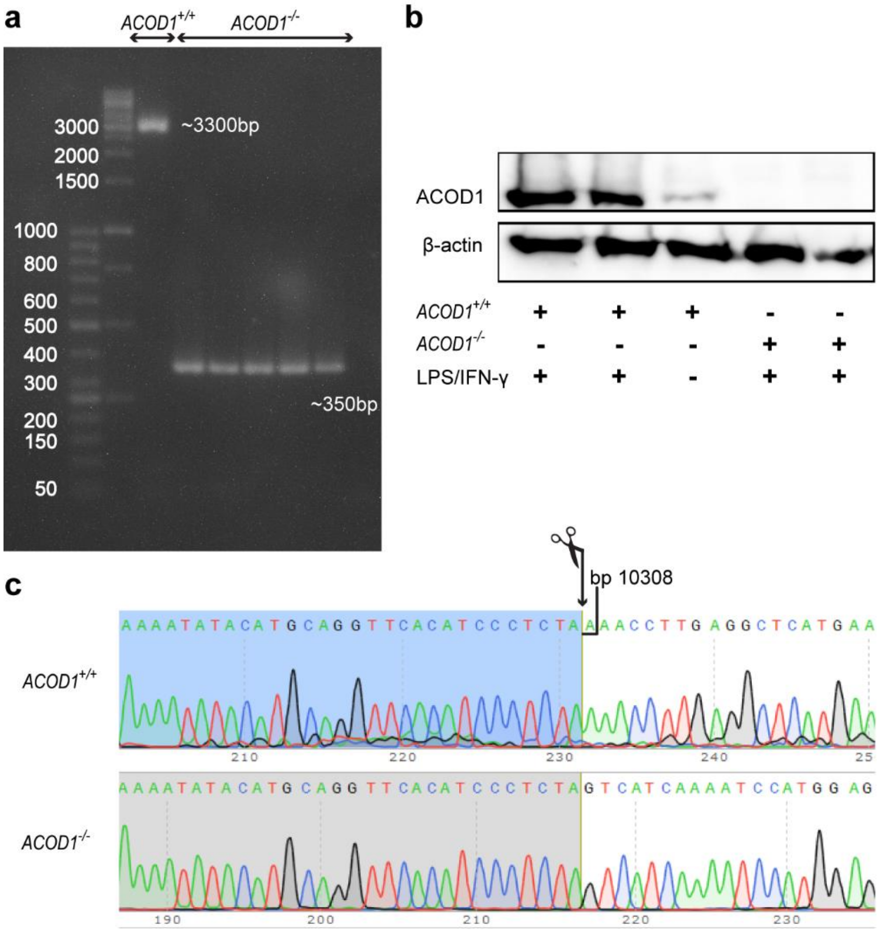

Figure S6. Verification of targeted deletion of *ACOD1* in THP1 cells. The *ACOD1* locus was inactivated using CRISPR/Cas9 technology by deleting a 2962 bp fragment spanning most of exon 4 and all of exon 5. **a**, Genomic PCR (5 clones) confirming the predicted 350 bp fragment expected in *ACOD1*$^{-/-}$ cells. **b**, Absence of ACOD1 protein in the *ACOD1*$^{-/-}$ cells before and after stimulation with LPS/IFN-γ. **c**, Verification of the sequence change in the 3' UTR upstream region at bp 10308 of the *ACOD1* gene (Sanger sequencing of lower strand). Shaded area, wild-type sequence. Analyses shown in **a** and **c** were performed once. The immunoblot shown in **b** is representative of 2 independent experiments.

Table S1. Quantum chemical descriptors of the itaconate isomers.[a]

Compound	E_{HOMO} (eV)	E_{LUMO} (eV)	Electronic chemical potential μ (eV)	Chemical hardness η (eV)	Electrophilicity ω (eV)	AUC Michael addition product
Itaconate	–0.9143	8.9889	4.0373	9.9032	0.82	96,139,619
Mesaconate	–0.9070	8.8985	3.9958	9.8055	0.81	18,724,894
Citraconate	–0.8703	9.2791	4.2044	10.1494	0.87	792,579,465

[a]E_{HOMO}: AM1-calculated energy of the highest occupied molecular orbital; E_{LUMO}: AM1-calculated energy of the lowest unoccupied molecular orbital; Electronic chemical potential calculated as $\mu = (E_{HOMO} + E_{LUMO})/2$; Chemical hardness in the ground state calculated as $\eta = E_{LUMO} - E_{HOMO}$; Global electrophilicity index calculated as $\omega = \mu^2/2\eta$.[3,4]

Table S2. Sequences of PCR primers used.

Gene name	Primer name	Sequence	Company
HPRT1	HPRT1-F	GAACGTCTTGCTCGAGATGTG	Eurofins
	HPRT1-R	CCAGCAGGTCAGCAAAGAATT	Eurofins
RPL27	RPL27-F	TGGACAAAACTGTCGTCAATAAGG	Eurofins
	RPL27-R	AGAACCACTTGTTCTTGCCTGTC	Eurofins
ACOD1	ACOD1-F	ATGCTGCTTTTGTGAACGGTG	Eurofins
	ACOD1-R	CTACCACGGAAGGGGGATGGA	Eurofins
CXCL10	CXCL10-F	CTGCTTTGGGGTTTATCAGA	Eurofins
	CXCL10-R	CCACTGAAAGAATTTGGGC	Eurofins
IL-6	IL-6-F	CTACATTTGCCGAAGAGCCC	Eurofins
	IL-6-R	CCCTGACCCAACCACAAATG	Eurofins
IL-1β	IL-1β-F	TACCCAAAGAAGAAGATGGAA	Eurofins
	IL-1β-R	GAGGTGCTGATGTACCAGTTG	Eurofins
TNFα	TNFα-F	ACCCTCTCTCCCCTGGAAAGGACA	Eurofins
	TNFα-R	TGAGGAACAAGCACCGCCTGGA	Eurofins
AKR1B10	AKR1B10-F	GAGGACCTGTTCATCGTCAGCA	Eurofins

	AKR1B10-R	CGTCCAGATAGCTCAGCTTCAG	Eurofins
SLC7A11	SLC7A11-F	TCCTGCTTTGGCTCCATGAACG	Eurofins
	SLC7A11-R	AGAGGAGTGTGCTTGCGGACAT	Eurofins
NQO1	NQO1-F	CCTGCCATTCTGAAAGGCTGGT	Eurofins
	NQO1-R	GTGGTGATGGAAAGCACTGCCT	Eurofins
HMOX1	HMOX1-F	CCAGGCAGAGAATGCTGAGTTC	Eurofins
	HMOX1-R	AAGACTGGGCTCTCCTTGTTGC	Eurofins
GCLC	GCLC-F	GGAAGTGGATGTGGACACCAGA	Eurofins
	GCLC-R	GCTTGTAGTCAGGATGGTTTGCG	Eurofins
GCLM	GCLM-F	TCTTGCCTCCTGCTGTGTGATG	Eurofins
	GCLM-R	TTGGAAACTTGCTTCAGAAAGCAG	Eurofins
GSS	GSS-F	CCAAGACCGAAGGCTGTTTGTG	Eurofins
	GSS-R	TGTGACCTCTCCAGCAGTAGAC	Eurofins
GSR	GSR-F	TATGTGAGCCGCCTGAATGCCA	Eurofins
	GSR-R	CACTGACCTCTATTGTGGGCTTG	Eurofins
TXNRD	TXNRD-F	GTTACTTGGGCATCCCTGGTGA	Eurofins
	TXNRD-R	CGCACTCCAAAGCGACATAGGA	Eurofins
GPX4	GPX4-F	ACAAGAACGGCTGCGTGGTGAA	Eurofins
	GPX4-R	GCCACACACTTGTGGAGCTAGA	Eurofins
ME1	ME1-F	GGAGTTGCTCTTGGTGTTGTGG	Eurofins
	ME1-R	GGATAAAGCCGACCCTCTTCCA	Eurofins
G6PD	G6PD-F	CTGTTCCGTGAGGACCAGATCT	Eurofins
	G6PD-R	TGAAGGTGAGGATAACGCAGGC	Eurofins
PGD	PGD-F	GTTCCAAGACACCGATGGCAAAC	Eurofins
	PGD-R	CACCGAGCAAAGACAGCTTCTC	Eurofins
IDH1	IDH1-F	CTATGATGGTGACGTGCAGTCG	Eurofins
	IDH1-R	CCTCTGCTTCTACTGTCTTGCC	Eurofins
HPRT1	Hs02800695_m1 (TaqMan probe, proprietary sequence)		ThermoFisher
AKR1B10	Hs00252524_m1 (TaqMan probe, proprietary sequence)		ThermoFisher

References (Supplement)

1 Sharma, P., Maklashina, E., Cecchini, G. & Iverson, T. M. The roles of SDHAF2 and dicarboxylate in covalent flavinylation of SDHA, the human complex II flavoprotein. *Proc Natl Acad Sci U S A* **117**, 23548-23556, doi:10.1073/pnas.2007391117 (2020).

2 Zhou, Q. *et al.* Thiabendazole inhibits ubiquinone reduction activity of mitochondrial respiratory complex II via a water molecule mediated binding feature. *Protein & cell* **2**, 531-542, doi:10.1007/s13238-011-1079-1 (2011).

3 Parr, R. G., Szentpály, L. v. & Liu, S. Electrophilicity Index. *J Am Chem Soc* **121**, 1922-1924, doi:10.1021/ja983494x (1999).

4 Zhou, Z. & Parr, R. G. Activation hardness: new index for describing the orientation of electrophilic aromatic substitution. *Am Chem Soc* **112, 5720-5724** (1990).
