# Supplementary material for: Citraconate inhibits ACOD1 (IRG1) catalysis, reduces interferon responses and oxidative stress, and modulates inflammation and cell metabolism
Source: Nat Metab. 2022 Jun 2;4(5):534–46. doi: 10.1038/s42255-022-00577-x (PMC9170585; doi:10.1038/s42255-022-00577-x)
Supplement: Supplementary file 2 — Reporting Summary [file 42255_2022_577_MOESM2_ESM.pdf]

nature portfolio

Corresponding author(s): Frank Pessler
Last updated by author(s): Apr 13, 2022

Reporting Summary

Nature Portfolio wishes to improve the reproducibility of the work that we publish. This form provides structure for consistency and transparency in reporting. For further information on Nature Portfolio policies, see our Editorial Policies and the Editorial Policy Checklist.

Statistics

For all statistical analyses, confirm that the following items are present in the figure legend, table legend, main text, or Methods section.

n/a	Confirmed	
☐	☒	The exact sample size (n) for each experimental group/condition, given as a discrete number and unit of measurement
☐	☒	A statement on whether measurements were taken from distinct samples or whether the same sample was measured repeatedly
☐	☒	The statistical test(s) used AND whether they are one- or two-sided<br>*Only common tests should be described solely by name; describe more complex techniques in the Methods section.*
☒	☐	A description of all covariates tested
☐	☒	A description of any assumptions or corrections, such as tests of normality and adjustment for multiple comparisons
☐	☒	A full description of the statistical parameters including central tendency (e.g. means) or other basic estimates (e.g. regression coefficient) AND variation (e.g. standard deviation) or associated estimates of uncertainty (e.g. confidence intervals)
☐	☒	For null hypothesis testing, the test statistic (e.g. F, t, r) with confidence intervals, effect sizes, degrees of freedom and P value noted<br>*Give P values as exact values whenever suitable.*
☒	☐	For Bayesian analysis, information on the choice of priors and Markov chain Monte Carlo settings
☒	☐	For hierarchical and complex designs, identification of the appropriate level for tests and full reporting of outcomes
☒	☐	Estimates of effect sizes (e.g. Cohen's d, Pearson's r), indicating how they were calculated

Our web collection on statistics for biologists contains articles on many of the points above.

Software and code

Policy information about availability of computer code

Data collection	No software was used for data collection.
Data analysis	GraphPad Prism 9.3.1. (GraphPad Software, San Diego, CA). Molecular Operating Environment (MOE), version 2020.09 (Chemical Computing Group ULC, 910–1010 Sherbrooke St. W. Montreal, Quebec, H3A 2R7, Canada). MetaboAnalyst 5.0, MetaboIndicator™ software (Biocrates Life Sciences, Innsbruck, Austria). Seahorse Wave desktop software 2.6 (Agilent, Waldbronn, Germany). Xcalibur 4.4.16.14, (OPTON-30965, Thermo Fisher, Langenselbold, Germany).

For manuscripts utilizing custom algorithms or software that are central to the research but not yet described in published literature, software must be made available to editors and reviewers. We strongly encourage code deposition in a community repository (e.g. GitHub). See the Nature Portfolio guidelines for submitting code & software for further information.

Data

Policy information about availability of data

All manuscripts must include a data availability statement. This statement should provide the following information, where applicable:

- Accession codes, unique identifiers, or web links for publicly available datasets
- A description of any restrictions on data availability
- For clinical datasets or third party data, please ensure that the statement adheres to our policy

Data availability
The following publicly accessible database was used: Protein Data Bank (PDB), with the following IDs: 6VAX (https://www.rcsb.org/structure/6VAX), 3SFD (https://www.rcsb.org/structure/3SFD), PDB ID: 6R6U (https://www.rcsb.org/structure/6R6U), 6R6T (https://www.rcsb.org/structure/6R6U). The raw data underlying the amino acids analyses and the multiplex cytokine chemokine analyses are included as Additional Supplemental Files (source data). All other data that support the

plots within this paper and other findings of this study are available from the corresponding author upon reasonable request.

Field-specific reporting

Please select the one below that is the best fit for your research. If you are not sure, read the appropriate sections before making your selection.

- [x] Life sciences
- [ ] Behavioural & social sciences
- [ ] Ecological, evolutionary & environmental sciences

For a reference copy of the document with all sections, see nature.com/documents/nr-reporting-summary-flat.pdf

Life sciences study design

All studies must disclose on these points even when the disclosure is negative.

Sample size

Sample sized were chosen based on previous experiments and experience and common usage in the research field of cellular metabolism. Since this was a new study, it was not possible to perform a pre-hoc power analysis. However, in our previously published studies on itaconate isomers and derivatives, similar experiments were conducted and very similar sample sizes (e.g., n=3 for cell-based assays) allowed for power sufficient to make similar conclusions about differences in abundance of itaconate isomers and of effects of exogenous application of itaconate and similar compounds on cell metabolism and inflammation. Winterhoff et al. 2021 (ref. 5 in the manuscript), Sohail et al. 2022 (ref. 28 in the manuscript).

Data exclusions

We did not exclude selected data. When an experiment was suspect of technical artefacts or produced negative data of no or only minor relevance to the overall interpretation of paper, the entire experiment was excluded.

Replication

Main manuscript:
Figure 1: A similar experiment had been performed as a pilot and yielded similar results.
Figure 2a-d: performed twice with similar results. e-h, Similar experiments had been performed in the lab twice before and yielded similar results, j-k were performed only once, but similar results regarding itaconate effect on pSTAT1 had been obtained in a previous experiment using itaconate as treatment (not the other isomers). l-n, actually is a compilation of 5 independent experiments (using lung tissue obtained from the surgeons on different dates) with n=3 tissue pieces per group each.
Figure 3: a-c, replicated once with reproducible results. d-e, performed once. f-h are modelling studies and replication is not applicable.
Figure 4: These experiments were performed only once, but the use of different doses and/or time points, showing a biologically plausible dose response and kinetics, provides additional robustness of the results.
Extended Data
Figure 1: similar uptake experiments had been performed twice, yielding very similar results.
Figure 2: a-h, performed once. i-l, had been performed twice before with very similar results.
Figure 3: see Figure 1, Main Manuscript.
Figure 4: a-c, the electrophilicity measurements were performed twice and yielded nearly identical results (15% variation, ranking was Citra>Ita>Mesa in both cases). e-h, are results of target-ligand modelling, and replication is not applicable. i, the experiment was performed once.
Figure 5: a-c, was performed twice, yielding similar results. d-g, was performed twice. In the repeat experiment (not shown here) the impact of the IAV infection was smaller, but the effects of the isomers were similar to the first experiment.
Figure 6: performed once, but f-q had been performed before using itaconate (but not mesaconate or citraconate), and the effects of itaconate were similar.
Figure 7: a-c, performed twice, yielding very similar results. d-f are results of target-ligand docking modelling and replication is not applicable.
Figure 8: performed once.

Supplement:
Figure S1: a-g, performed once. h, MTT assays with the isomers had been performed before at least once with THP1 cells, but also with A549 cells (not included here), yielding similar results.
Figure S2: a-d, performed once. e-g are results of target-ligand modelling studies and replication is not applicable.
Figure S3: see Extended Data Figure 5.
Figure S4: see Extended Data Figure 5.
Figure S5: performed once.
Figure S6: a,c ,performed once. b, performed twice. The selected ACOD1-/- clone has maintained its phenotype throughout the experiments.

Randomization

Experimental groups were randomized only to the extent that the sample groups were distributed across 96-well plates, as indicated, in order to avoid positional artefacts such as edge effects.

Blinding

The samples were not blinded in order to minimize the risk of misidentifying samples throughout the course the experiment. Blinding was not necessary because data were generated from cells or organs and all analyses were performed using objective, standardized equipment and assay kits (RT-qPCR, immunoassay, mass spectrometry etc.). Analyses that could have been affected by preconceived expections on part of the examiner (e.g., scoring of unblinded histological slides) were not part of this study.

Reporting for specific materials, systems and methods

We require information from authors about some types of materials, experimental systems and methods used in many studies. Here, indicate whether each material, system or method listed is relevant to your study. If you are not sure if a list item applies to your research, read the appropriate section before selecting a response.

Materials & experimental systems

n/a	Involved in the study
☐	☒ Antibodies
☐	☒ Eukaryotic cell lines
☒	☐ Palaeontology and archaeology
☐	☒ Animals and other organisms
☐	☒ Human research participants
☒	☐ Clinical data
☒	☐ Dual use research of concern

Methods

n/a	Involved in the study
☒	☐ ChIP-seq
☒	☐ Flow cytometry
☒	☐ MRI-based neuroimaging

Antibodies

Antibodies used

STAT1 (Santa Cruz Biotechnology, sc-345, diluted 1:500), phospho-STAT1 (Cell Signaling, 9167S, diluted 1:1000), NRF2 (Cell Signalling, 12721S, diluted 1:1000). Goat anti-rabbit IgG-HRP (Southern Biotech, 4030-05). β-actin was visualized using HRP-conjugated anti-β-actin antibody (Abcam; ab49900) or β-actin antibody (C4) mouse mAb IgG1 (Santa Cruz Biotechnology, sc-47778). Anti-IAV-NP mouse monoclonal IgG (provided as hybridoma supernatant from Prof. Stephan Ludwig, Münster, Germany; diluted 1:100). Goat anti-mouse IgG (H+L) HRP-conjugated secondary antibody (Invitrogen; A16072), (diluted 1:1000).

Validation

All antibodies, except anti-IAV-NP, were from commercial sources and had been validated by the manufacturers.
anti-STAT1 (Santa Cruz Biotechnology, sc-345, diluted 1:500): by western blot analysis, i.e. recognition of the band of the expected migration, using human, mouse and rat cell lysates.
anti-phospho-STAT1 (Cell Signaling, 9167S): by western blot analysis of extracts from HeLa cells untreated or treated with interferon-α (IFN-α), showing induction of the two expected isoform bands by IFNa in HeLa cells.
anti-NRF2 (Cell Signalling, 12721S): by western blot analysis of extracts from MEF wt and U-2 OS cells, untreated (-) or treated with MG-132 #2194 (10 µM, 10 hr).
Goat anti-rabbit IgG-HRP (Southern Biotech, 4030-05): by dose-response using ELISA coated with purified rabbit IgG and IgM.
HRP-conjugated anti-β-actin antibody (Abcam; ab49900): by western blot analysis using lysates of multiple cell lines from various species including human, recognizing the single band of the expected migration of 42 kDa.
β-actin antibody (C4) mouse mAb IgG1 (Santa Cruz Biotechnology, sc-47778): by fluorescent western blot analysis of β-Actin expression in Jurkat (A), HeLa (B) and A-431 (C) whole cell lysates, revealing the specific band of the expected migration of 42 kDa.
Anti-IAV-NP mouse monoclonal IgG: in-cell histochemistry and immunofluorescence comparing IAV-infected against uninfected cells.
Goat anti-mouse IgG (H+L) HRP-conjugated secondary antibody (Invitrogen; A16072): western blot analysis on whole cell extracts (30 µg lysate) of K-562 and HEL 92.1.7 cells, revealing the specific band of the expected migration of about 85 kD.
We additionally verified performance of all primary antibodies, except anti-NP, by western blot analysis by recognizing the specific band of the expected migration in SDS-PAGE and including negative and positive control samples as indicated.

Eukaryotic cell lines

Policy information about cell lines

Cell line source(s)

THP1 cells: German Collection of Microorganisms and Cell Cultures GmbH (DSMZ) no. ACC 16. A549 cells: DSMZ, no. ACC107.
HaCaT kindly provided by Thomas Werfel (Hannover Medical School, Hannover, Germany).
MDCK-II: American Tissue Culture Collection (ATCC) no. CRL-2936.

Authentication

THP1 had been authenticated by the provider (DSMZ), using (i) STR analysis according to the global standard ANSI/ATCC ASN-0002.1-2021 (2021) which resulted in an authentic STR profile of the reference STR database and (ii) cytogenetics, revealing human near-tetraploid karyotype - 94(88-96)<4n>XY/XXY, -Y, +1, +3, +6, +6, -8, -13, -19, -22, -22, +2mar, add(1)(p11), del(1)(q42.2), i(2q), del(6)(p21)x2-4, i(7p), der(9)t(9;11)(p22;q23)i(9)(p10)x2, der(11)t(9;11)(p22;q23)x2, add(12)(q24)x1-2, der(13)t(8;13)(p11;p12), add(?18)(q21) - carries t(9;11) associated with AML M5.
A549: The cell lines had been authenticated by the provider (DSMZ) by STR analysis according to the global standard ANSI/ATCC ASN-0002.1-2021 (2021) resulted in an authentic STR profile of the reference STR database. And by cytogenetics: human hypotriploid karyotype with 8% polyploidy - 65(59-66)<3n>XXY, -1, -3, -6, +12, -13, -15, -18, -19, -21, -22, +4mar, der(6)t(1;6)(q11;q27), del(11)(q22-23) - presence of large distinctive der(6) marker confirms identity of this cell line.
HaCaT: characteristic ability to form stratified epithelium and desmosomes in a 3D epidermis model in co-culture with immortalized fibroblasts, expression of typical cell surface marker, desmoglein 1, in this model (Malik, Waqas et al. J Clin Invest 2021).
MDCK-II American Tissue Culture Collection (ATCC) no. CRL-2936. Surface markers: E-cadherin (epithelial cell adhesion molecule), expressed; Zona Occludens (ZO-1) (tight junction protein), expressed; fibroblast-specific protein (FSP), not expressed; cytokeratin (CK1, 4, 5, 6, 8, 10, 13, 18, 19), expressed; sialic receptors: alpha 2,3-galactose (avian) and alpha 2,6-galactose (human), expressed. Gene expression: zona occludens (ZO-1); E-cadherin ; fibroblast-specific protein (FSP) not expressed; cytokeratins: (CK1; CK4; CK5; CK6; CK8; CK10; CK13; CK18; CK19); sialic receptor alpha 2,3-galactose (avian); sialic

	receptor alpha 2,6-galactose (human).
Mycoplasma contamination	The cell lines were regularly tested for Mycoplasma contamination and only negative cultures were used for the experiments.
Commonly misidentified lines (See ICLAC register)	No commonly misidentified lines were used.

Animals and other organisms

Policy information about studies involving animals; ARRIVE guidelines recommended for reporting animal research

Laboratory animals	C57BL/6N male and female mice, 3-4 month-old, obtained from Charles River Laboratories (France)
Wild animals	No wild animals were used.
Field-collected samples	No field-collected samples were used.
Ethics oversight	All animal procedures were approved by the University of Luxembourg Animal Experimentation Ethics Committee and by appropriate government agencies. The animal work of the present study has been conducted and reported in accordance with the ARRIVE (Animal Research: Reporting of in vivo Experiments) guidelines to improve the design, analysis and reporting of research using animals, maximizing information published and minimizing unnecessary studies.

Note that full information on the approval of the study protocol must also be provided in the manuscript.

Human research participants

Policy information about studies involving human research participants

Population characteristics	For the human lung model, explanted human lung tissue (i.e. lung that was from a patient who then received a lung transplant) was provided by the Dept. of Pathology, Hannover Medical School. The underlying diagnoses were emphysema (n=3) and idiopathic pulmonary fibrosis (n=2), male = 2, female = 3, age 58-66 y.
Recruitment	The samples were obtained as part of an ongoing pipeline between the Dept. of Cardiothoracic Surgery and Dept. of Pathology of Hannover Medical School, where all explanted lungs are checked by Pathology for suitability for research use and, if deemed suitable, are shared with several research groups on the campus.
Ethics oversight	Use of human tissues was approved by the Ethics Committee of Hannover Medical School (file no. 2923-2015), and all donors gave informed consent before the surgery for use of tissue for research purposes.

Note that full information on the approval of the study protocol must also be provided in the manuscript.
