# Supplementary figures and images for: Citraconate inhibits ACOD1 (IRG1) catalysis, reduces interferon responses and oxidative stress, and modulates inflammation and cell metabolism
Source: Nat Metab. 2022 Jun 2;4(5):534–46. doi: 10.1038/s42255-022-00577-x (PMC9170585; doi:10.1038/s42255-022-00577-x)

**Fig. S6b (dTHP1 cells)**

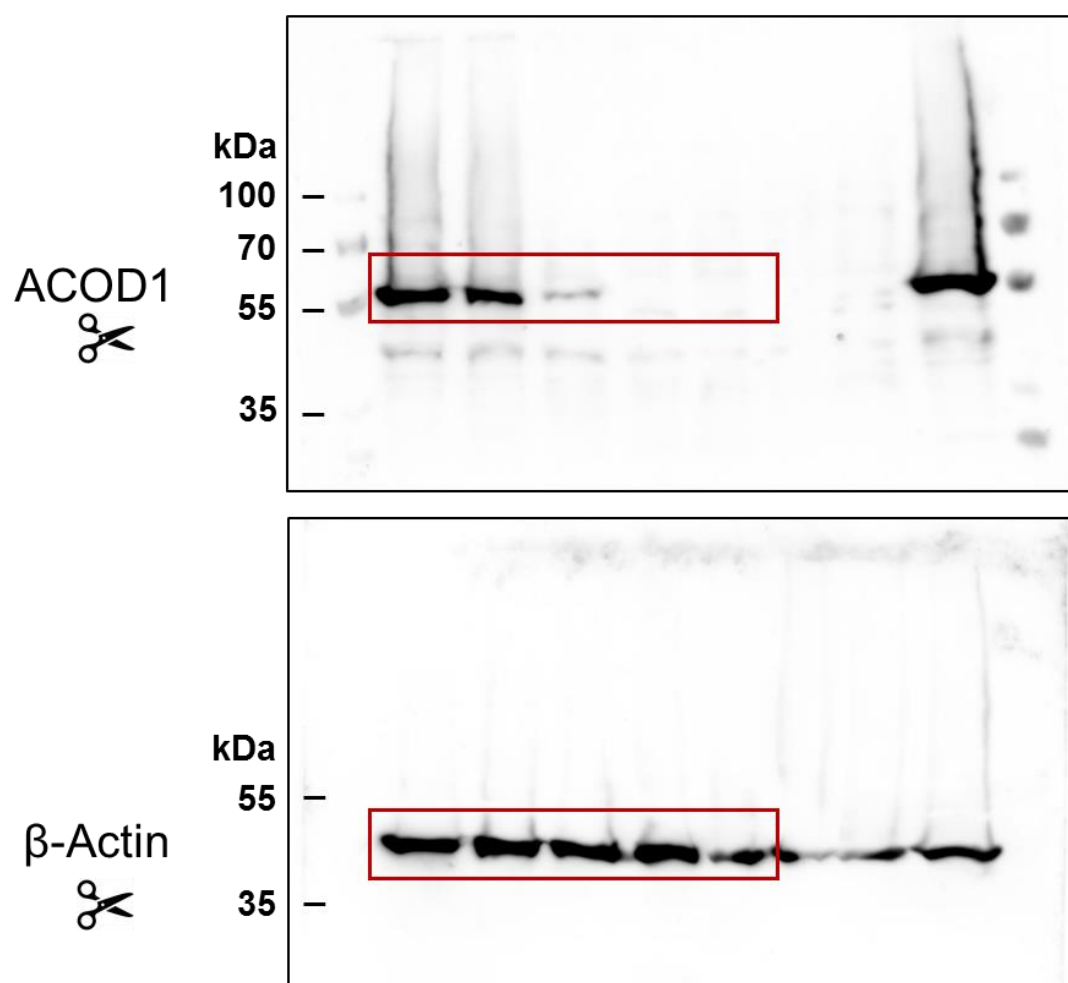

Supplement: Supplementary file 5 — Source data blots for Supplementary Fig. 6b. [file 42255_2022_577_MOESM5_ESM.pdf]

**Fig. 2a (HaCaT cells)**

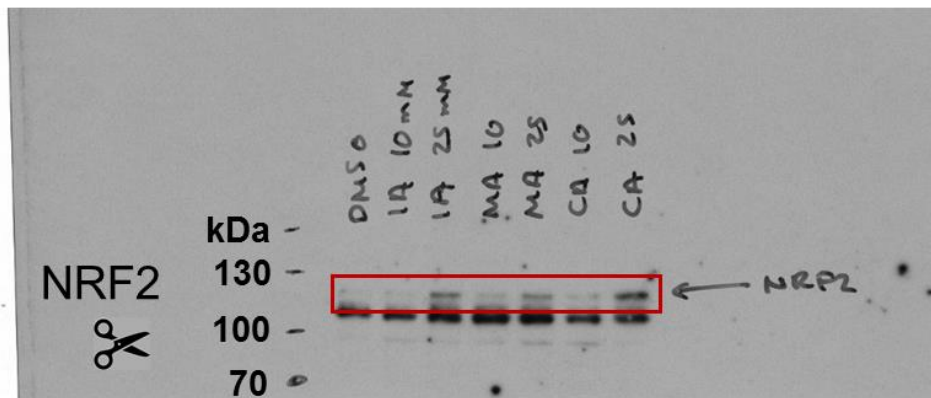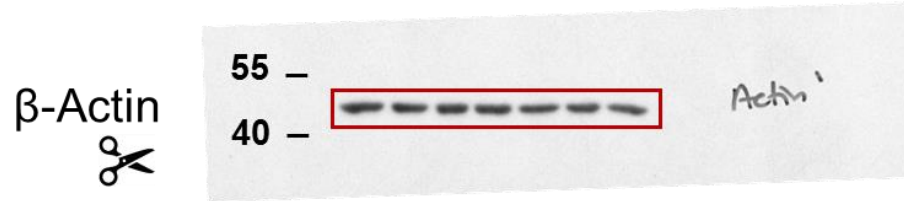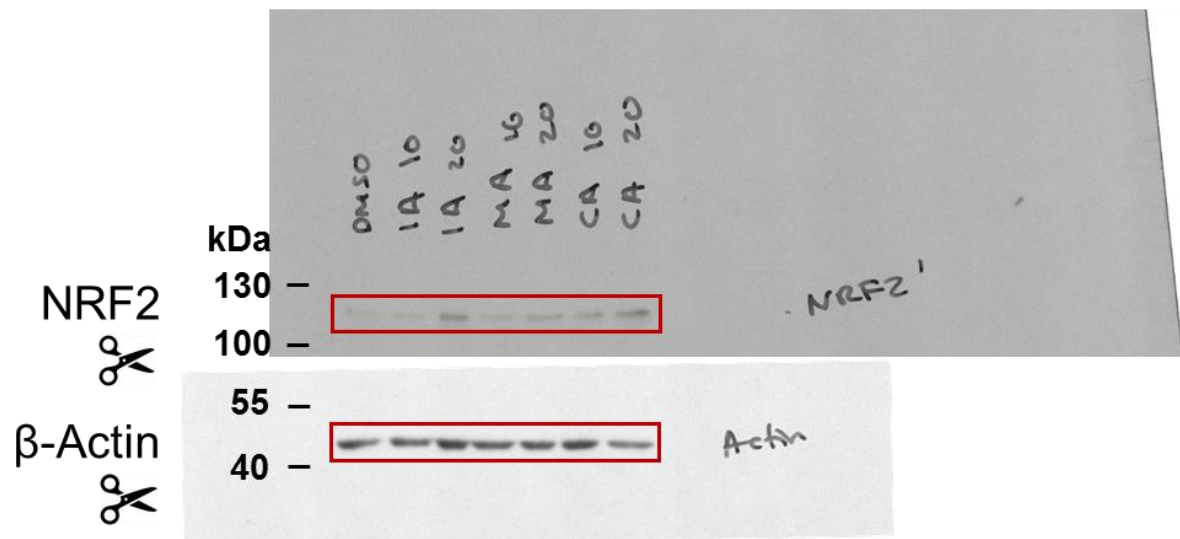

**Fig. 2j (dTHP1 cells)**

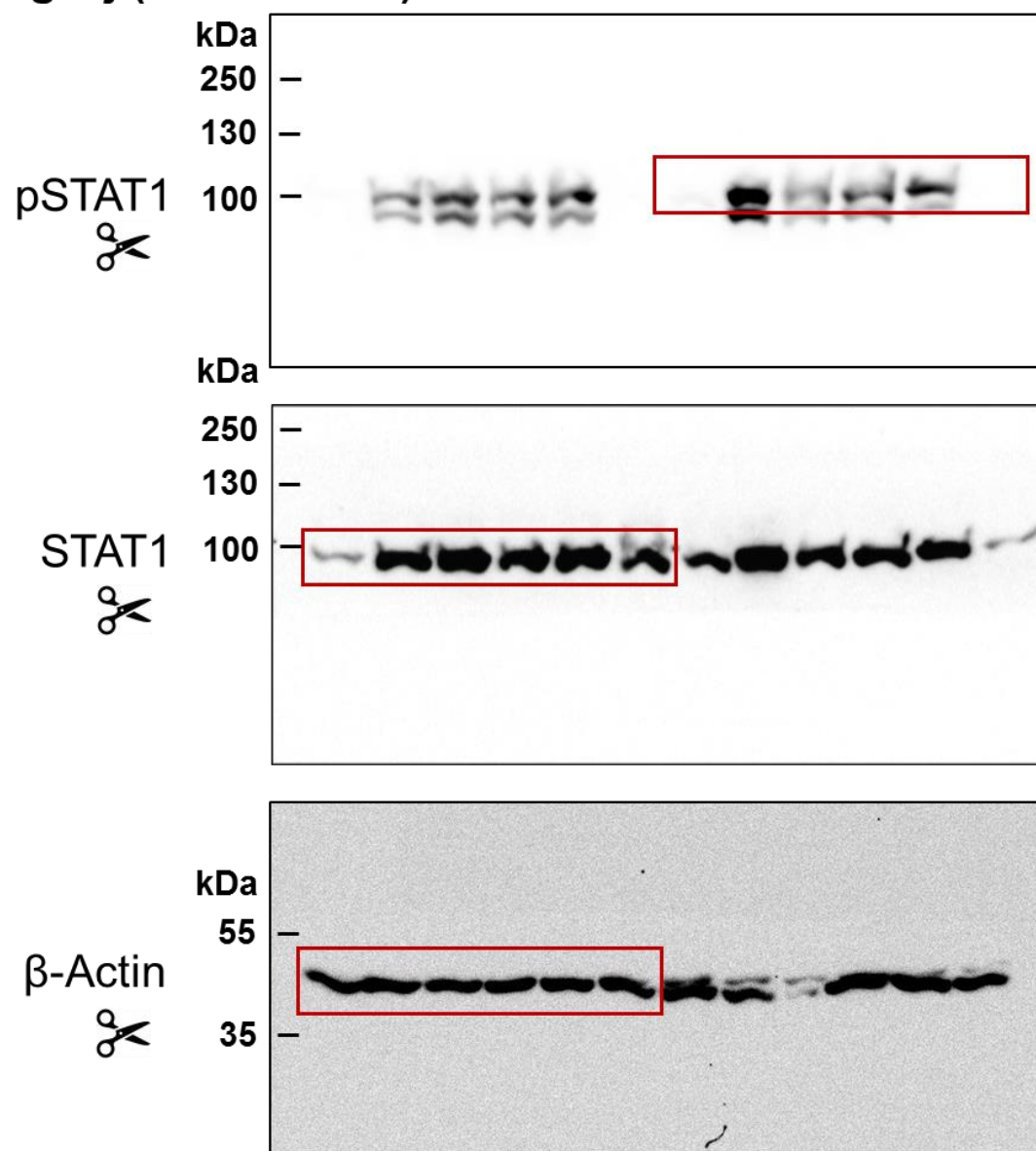

**Fig. 2k (A549 cells)**

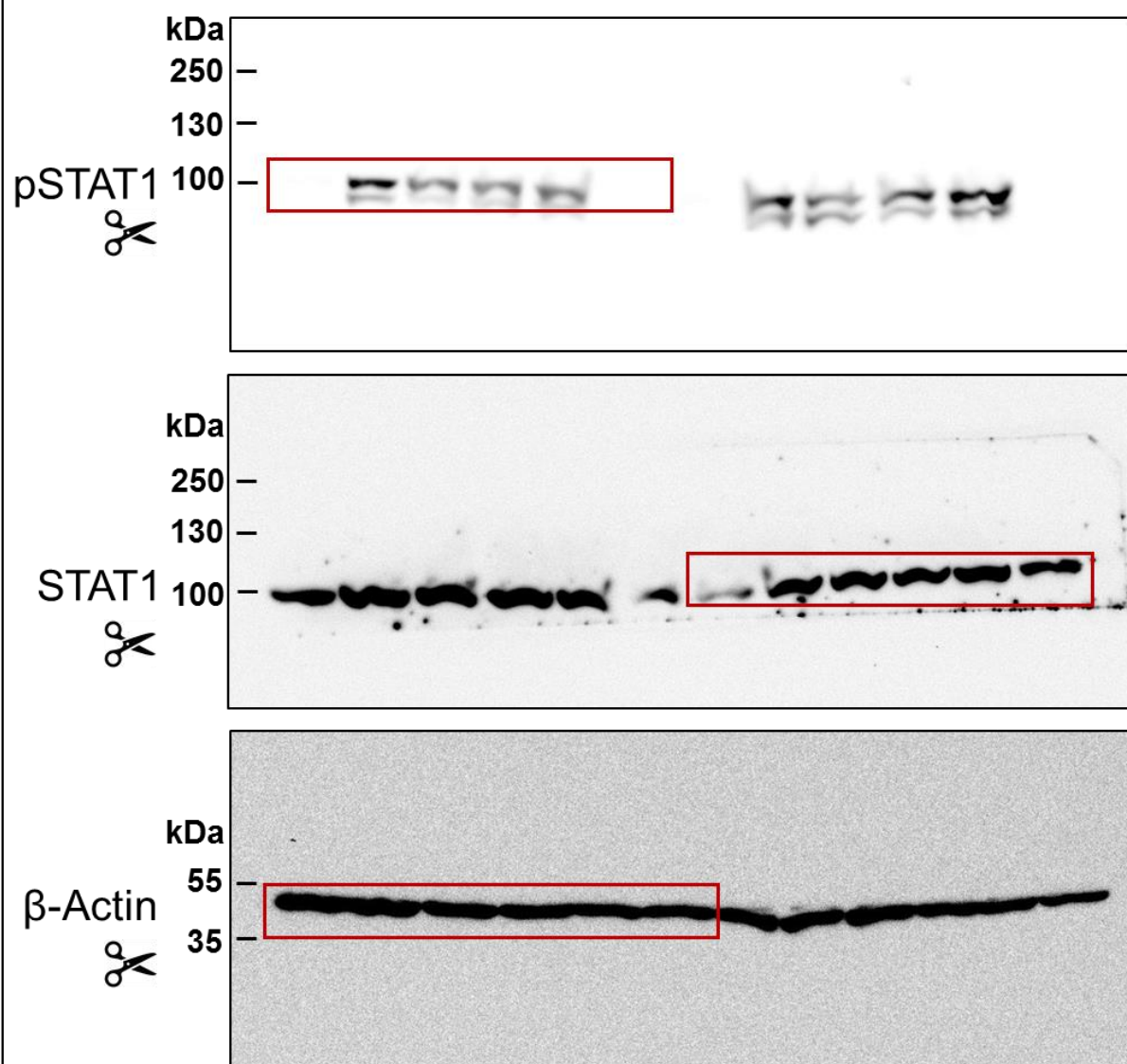

Supplement: Supplementary file 6 — Uncropped membrane images of immunoblots, Fig. 2a, j and k. [file 42255_2022_577_MOESM6_ESM.pdf]
